# Photochromism‐Driven Full Solar Spectrum Absorption for Efficient Photo‐Thermo‐Electric Conversion

**DOI:** 10.1002/advs.202518602

**Published:** 2025-11-27

**Authors:** Ning‐Ning Zhang, Lin‐Xu Liu, Zhen‐Yu Li, Yong‐Fang Han, Wen‐Wen Zi, Kong‐Gang Qu, Ming‐Sheng Wang, Yong Yan

**Affiliations:** ^1^ School of Chemistry and Chemical Engineering Liaocheng University Liaocheng Shandong 252000 P. R. China; ^2^ State Key Laboratory of Structural Chemistry Fujian Institute of Research on the Structure of Matter Chinese Academy of Sciences Fuzhou Fujian 350108 P. R. China; ^3^ School of Environmental and Materials Engineering Yantai University Yantai Shandong 264005 P. R. China; ^4^ Fujian Key Laboratory of Advanced Inorganic Oxygenated Materials Fuzhou University Fuzhou Fujian 350108 P. R. China

**Keywords:** electron‐transfer photochromism, full solar spectrum absorption, NIR photothermal effect, photo‐thermo‐electric conversion

## Abstract

Full solar spectrum absorbing photothermal materials may maximally utilize solar energy in emerging photo‐thermo‐electric conversion technology for outdoor energy harvesting, remote power supply, wearable electronics, and aerospace. However, composite materials with full solar spectrum absorption still suffer from low open‐circuit voltages (≤ 200 mV) and limited power densities (≤1 W·m^−2^), and single‐component materials are subject to insufficient absorption range (< 1100 nm), complicated synthesis process, or expensive precursors. Herein, a readily available viologen‐based organic compound with a π‐stacked supramolecular structure is found to exhibit full solar spectrum absorption (250–2500 nm) and high near‐infrared (NIR) photothermal conversion efficiency (PCE >60%) after electron‐transfer photochromism. Integrated with a commercial thermoelectric generator (TEC1‐12701), the resulting device achieved an open‐circuit voltage of 292.3 mV and a power density of 1.30 W·m^−2^ under 1 Sun irradiation. This photochromic strategy provides a new path to achieve efficient photo‐thermo‐electric conversion.

## Graphical abstract

After electron‐transfer photochromism, a black viologen‐based organic compound shows full solar spectrum absorption (250–2500 nm) and near‐infrared photothermal conversion efficiency (NIR‐PCE) >60%. Integrated with a thermoelectric generator, it outputs 1.17 V under 4 Suns irradiation, enough to drive a small fan via photo‐thermo‐electric conversion.

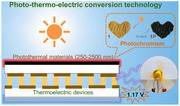

## 1 Introduction

**Scheme 1:**
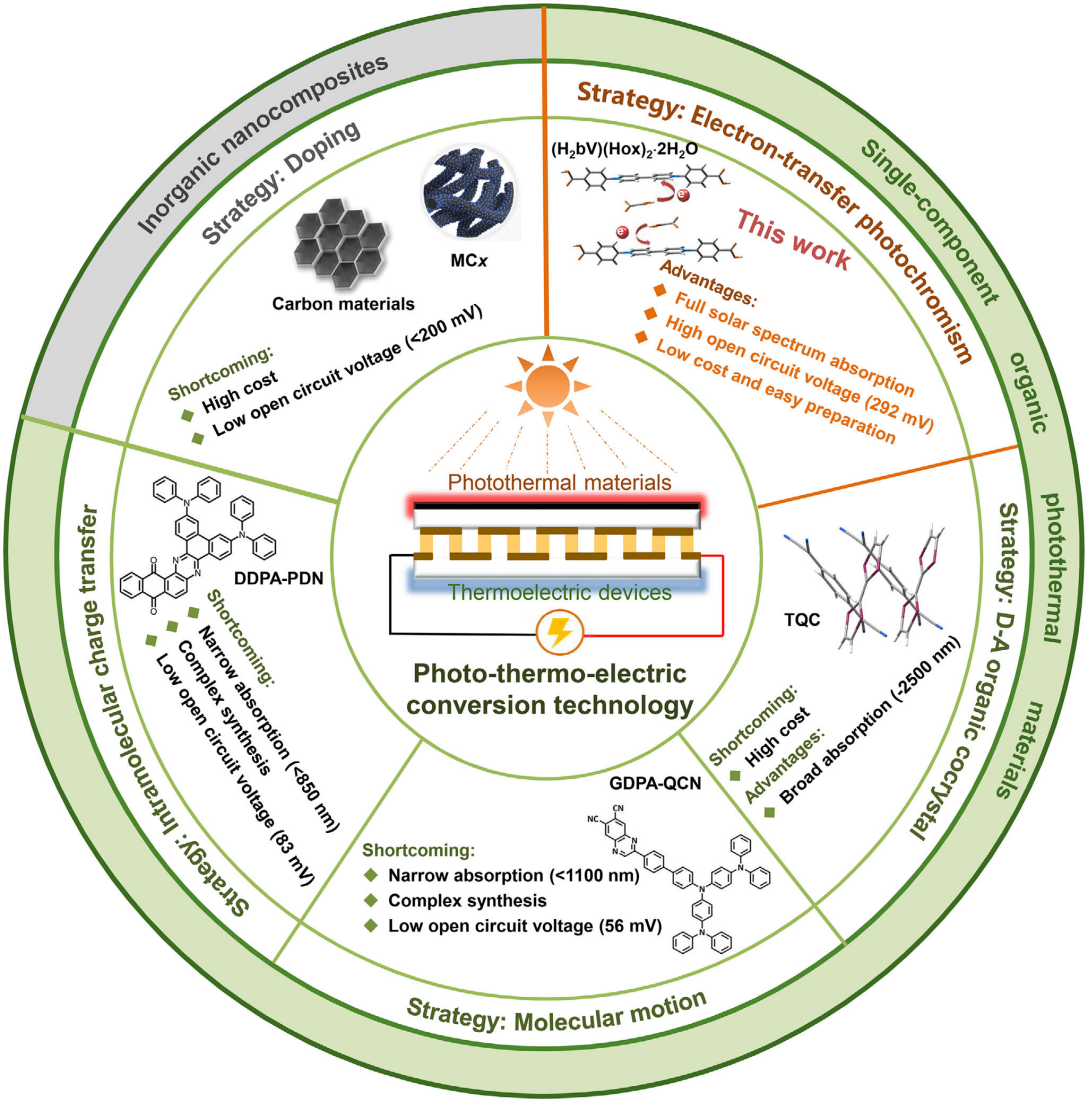
Advantages, disadvantages, and strategies of different photothermal materials for photo‐thermo‐electric conversion technology.

Amid escalating energy and environmental challenges, solar energy emerges as a clean and abundant alternative.^[^ ^1^ ^]^ Photo‐thermo‐electric conversion,^[^ ^2^, ^3^ ^]^ which integrates photothermal and thermoelectric processes,^[^ ^4^, ^5^ ^]^ offers a promising technology for solar energy harvesting in applications such as outdoor power, remote supply, wearables, and aerospace.^[^ ^6^ ^]^ In photo‐thermo‐electric devices, photothermal materials absorb sunlight and convert it into heat, generating a temperature gradient that drives electricity via the Seebeck effect.^[^ ^7^, ^8^ ^]^ To maximize the conversion of solar energy into electricity, photothermal materials must possess both broad spectral absorption, spanning from the ultraviolet to the infrared region, and high photothermal conversion efficiency (PCE).^[^ ^9^, ^10^ ^]^ In particular, photothermal materials with full solar spectrum absorption (280–2500 nm)^[^ ^11^ ^]^ are crucial, as the near‐infrared (NIR) region alone accounts for over 53% of the total solar energy. Existing full solar spectrum absorbing photothermal materials are primarily based on metal‐,^[^ ^12^, ^13^, ^14^ ^]^ carbon‐,^[^ ^15^, ^16^ ^]^ or polymer‐based^[^ ^17^ ^]^ nanocomposites, which typically suffer from low open‐circuit voltages (≤200 mV) and limited power densities (≤ 1 W·m^−2^) (Table S1, Supporting Information). Recently, single‐component organic photothermal materials have garnered growing attention due to their tunable structures, facile synthesis, and efficient energy conversion.^[^ ^18^, ^19^ ^]^ A common approach to broaden their absorption spectrum is often constructing polycyclic π‐conjugated systems, such as 2,17‐bis(diphenylamino)dibenzo[a,c]naphtho[2,3‐h]phenazine‐8,13‐dione (DDPA–PDN)^[^ ^20^ ^]^ (< 850 nm) and (dendritic triphenylamine)‐phenyl–quinoxaline‐6,7‐dicarbonitrile (GDPA–QCN)^[^ ^21^ ^]^ (< 1100 nm). However, they require complex and time‐consuming synthetic routes (**Scheme** **1**), aside from the insufficient absorption range. Although intramolecular charge transfer (ICT)^[^ ^20^ ^]^ and molecular motion (MM)^[^ ^21^ ^]^ strategies enhance PCE, the open‐circuit voltages of their photo‐thermo‐electric devices remain very unsatisfactory low (Scheme 1). A notable advance by Li et al.^[^ ^6^ ^]^ employed a donor–acceptor organic cocrystal (TQC) to achieve full solar spectrum absorption with improved voltage output, but its dependence on costly components like tetrathiafulvalene (TTF) hinders practical deployment (Scheme 1). These limitations underscore the urgent need for more versatile and efficient molecular design strategies to realize organic photothermal materials with full solar spectrum absorption and efficient photo‐thermo‐electric conversion.

Photochromic compounds, as a class of smart stimuli‐responsive materials, undergo reversible color changes and display broadened absorption spectra under electromagnetic radiation.^[^ ^22^ ^]^ These features enable their application in anti‐counterfeiting,^[^ ^23^, ^24^ ^]^ catalysis,^[^ ^25^, ^26^ ^]^ separation,^[^ ^27^, ^28^ ^]^ optical switches,^[^ ^29^, ^30^, ^31^ ^]^ and smart windows.^[^ ^32^ ^]^ Among them, electron‐transfer photochromic compounds^[^ ^33^ ^]^ are particularly promising for solid‐state applications, owing to their minimal structural changes upon light stimulation. The photogenerated radicals^[^ ^34^ ^]^ and their π‐aggregates exhibit broad optical absorption,^[^ ^35^, ^36^, ^37^, ^38^ ^]^ potentially covering the entire solar spectrum.^[^ ^39^, ^40^ ^]^ Moreover, π‐stacking enhances radical stability, suppresses radiative decay, and promotes non‐radiative relaxation, thereby improving the PCE.^[^ ^41^, ^42^ ^]^ These advantages render electron‐transfer photochromic systems ideal candidates for full solar spectrum absorbing organic photothermal materials in photo‐thermo‐electric devices.

In this work, we report the first application of photochromism in photo‐thermo‐electric conversion. A viologen‐based photochromic organic salt, [(H_2_bV)(Hox)_2_·2H_2_O] (**1**), was successfully synthesized via a solvothermal reaction, in which H_2_bV (1,1′‐bis(2,4‐carboxyphenyl)‐4,4′‐bipyridinium) and Hox (mono‐deprotonated oxalate) act as electron acceptor and donor, respectively. Upon Xe lamp irradiation, compound **1** undergoes a distinct color change from yellow to black (**1P**), along with the emergence of the full solar spectrum absorption (250–2500 nm) and a high NIR photothermal conversion efficiency exceeding 60%. Theoretical calculations attribute these outstanding properties to strong π‐stacking interactions between Hox^•^ and H_2_bV^•^ radicals. When integrated **1P** samples into a thermoelectric device (**1P**@TEC1‐12701), the system delivers an open‐circuit voltage of 292.3 mV and a power density of 1.30 W·m^−2^ under 1 Sun irradiation. This work establishes a new strategy for coupling photochromism with efficient photo‐thermo‐electric conversion (Scheme 1).

## 2 Results and Discussion

### 2.1 Crystal Structure

**Figure 1:**
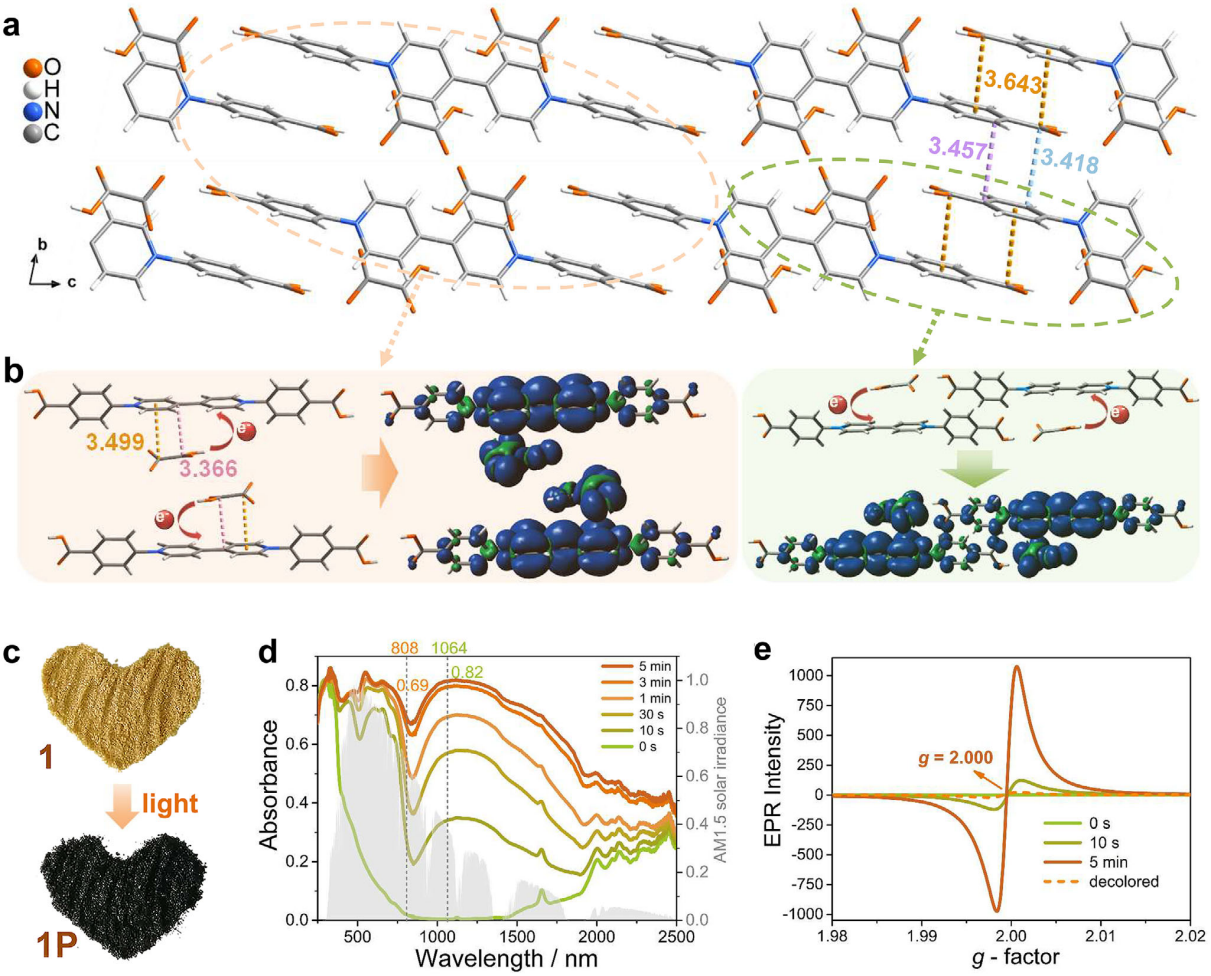
a) 2D hydrogen‐bonded framework stacked along the a axis, and intermolecular *π* interactions between adjacent H_2_bV ligands. b) Intermolecular π interactions between Hox and H_2_bV ligands. And spin density of 2[(Hox^•^)(H_2_bV^•^)]‐b and 2[(Hox^•^)(H_2_bV^•^)]‐a, respectively. c) Sample pictures of **1** and **1P**, respectively. d) Time‐dependent solid‐state absorption spectra (gray spectrum: the AM 1.5 solar spectral irradiance). e) Time‐dependent solid‐state EPR spectra. Decolored: the data for the irradiated saturated samples after 75 days of storage in the dark at room temperature. Note: All separations are labeled in angstroms.

Compound **1** was synthesized as a phase‐pure material by a step solvothermal reaction (detailed procedures are provided in the Figures S1 and S2, Supporting Information). Single‐crystal X‐ray diffraction (SC‐XRD) analysis shows that **1** crystallizes in the triclinic space group *P* $\bar{1}$ with two formula units per unit cell. Each asymmetric unit consists of half of a H_2_bV cation, a Hox anion, and a lattice water molecule (Figure S3, Supporting Information). The mono‐deprotonated oxalate unit serves as a bridge, linking half of the H_2_bV ligands and three lattice water molecules through four distinct hydrogen bonds, with bond lengths spanning from 1.751 to 1.951 Å (Figure S3, Supporting Information). These interactions construct a 2D hydrogen‐bonded network, with the H_2_bV ligands anchoring clusters of oxalic acid and water molecules in a layered arrangement (Figure S4, Supporting Information). These 2D hydrogen‐bonded layers assemble into a 3D dense structure by repeated slip‐stacking (**Figure** **1**a). Notably, intermolecular π interactions occur both between Hox and H_2_bV ligands and among adjacent H_2_bV ligands (Figure S5, Supporting Information). Specifically, for the *π*–*π* interactions, the distance from the carbonyl carbon atoms to the centers of the phenyl rings is 3.643 Å (Figure 1a); the distance between the phenyl carbon atoms is ≈3.457 Å, and the distance from the carbonyl carbon atoms to the phenyl carbon atoms is 3.418 Å. For the *p*–*π* interactions, the distance between the oxalic acid carbon atom and the pyridine ring centroid is ≈3.499 Å (Figure 1b), while the distance from the oxalic acid carbon atom to the pyridine carbon atom is 3.366 Å.

### 2.2 Photochromic Property

Compound **1** exhibits rapid and excellent photochromism, changing from yellow to green within 10 s, dark green after 30 s, and fully black after 5 min of xenon lamp irradiation (Figure 1c; Figure S6, Supporting Information). The saturated state, **1P**, shows significantly enhanced absorption spanning 300–2500 nm, with initial changes in the visible region (300–750 nm) and subsequent growth in the NIR region (750–2500 nm) (Figure 1d). Electron paramagnetic resonance (EPR) measurements confirm that the photochromic behavior arises from the generation of free radicals (Figure 1e). These radicals remain stable for at least 75 days in the dark at room temperature (Figure 1e) and are unaffected by heating at 120 °C for 2 h (Figure S7, Supporting Information), indicating excellent air and thermal stability.

To explore the origin of the full solar spectrum absorption of **1P**, we conducted structural analysis and theoretical calculations. Structurally, compound **1** contains one electron acceptor (4,4′‐bipyridinium moiety) and two potential electron donors (benzoic group and the Hox anion), but the dominant donor could not be identified from the structure alone. Density of states (DOS) and partial DOS (PDOS) analyses indicated that the Γ_a_, Γ_b,_ and Γ_c_ states primarily arise from the contribution of *p* orbitals of Hox (Figure S8a,c, Supporting Information), while the Γ_d_ states originate from the antibonding *p_π_* orbitals of 4,4′‐bipyridinium moieties in H_2_bV (Figure S8a,b, Supporting Information), confirming that electron transfer occurs primarily from Hox to H_2_bV. To further evaluate the contributions of Hox^•^ and H_2_bV^•^ radicals to the broad absorption spectrum, time‐dependent density functional theory (TD‐DFT) calculations of optical absorption were conducted for a series of radical species using the B3LYP/6‐311G(d,p) level. The monomeric radical (H_2_bV^•^) and dimeric radicals ([2H_2_bV^•^], 2[H_2_bV^•^]) exhibited optical absorption only in the UV–vis range (250–750 nm) (Figure S9a–c, Supporting Information), indicating their role in short‐wavelength absorption. In contrast, introducing Hox^•^ into the model ([Hox^•^⋅H_2_bV^•^]) led to significant NIR absorption (Figure S9d, Supporting Information). Further π‐stacking between Hox^•^ and H_2_bV^•^ radicals, as modeled in 2[Hox^•^⋅H_2_bV^•^]‐a and 2[Hox^•^⋅H_2_bV^•^]‐b, resulted in pronounced red‐shifted absorption bands extending beyond 2000 nm (Figure S9e,f, Supporting Information), accounting for the broad NIR absorption (750–2500 nm) observed experimentally. Spin density analysis further supports this, showing delocalized spin populations across both 4,4′‐bipyridinium moieties of H_2_bV and Hox units in the stacked radical states (Figure 1b). These results collectively indicate that the UV–Vis absorption (300–750 nm) of **1P** originates from H_2_bV^•^ radicals, while the broad NIR absorption (750–2500 nm) arises from π‐stacked Hox^•^–H_2_bV^•^ radical pairs.

### 2.3 NIR Photothermal Effects

**Figure 2:**
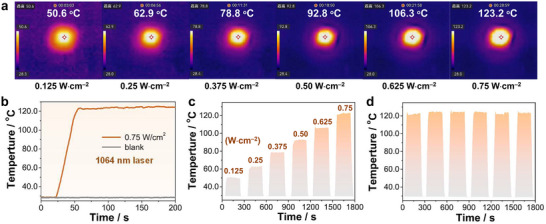
a) Infrared photos of a crystalline pellet of **1P** under different irradiation power densities of 1064 nm laser. b) Temperature curves of **1P** and blank quartz glass plate under the irradiation of 0.75 W cm^−2^ 1064 nm laser. c) Temperature changes of **1P** under the irradiation of a 1064 nm laser with different power densities. d) Cycling temperature curve of **1P** under the irradiation of 1064 nm laser with a specific power density of 0.75 W cm^−2^.

Under 808 nm (0.75 W·cm^−2^) and 1064 nm (0.75 W·cm^−2^) laser irradiation, the temperature of **1P** crystalline pellets rapidly increased from 27 °C and 28 °C to ≈84.5 °C (Figure S10a,b, Supporting Information) and 123.2 °C (**Figure** **2**a,b), respectively. In contrast, no obvious temperature increase was observed for a blank quartz glass plate under identical conditions (Figure 2b; Figure S10b, Supporting Information). When the 808 nm laser power was varied from 0.125 to 0.75 W·cm^−2^, the pellet temperature increased linearly from 39.9 °C to 84.5 °C, with corresponding Δ*T*_max_ values of 13.1 to 57.8 °C (Figure S10a,c, Supporting Information). Similarly, under 1064 nm irradiation, Δ*T*_max_ values ranged from 22.3 to 95.2 °C over the same power range (Figure 2a,c), confirming robust NIR‐I and NIR‐II photothermal responsiveness. Photothermal cycling tests under both wavelengths at 0.75 W·cm^−2^ showed consistent heating profiles over six cycles (Figure 2d; Figure S10d, Supporting Information), indicating excellent photothermal stability. Moreover, the 6‐h continuous irradiation and 60 on/off cycles deeply demonstrate the splendid photostability of the **1P** pellet (Figure S11, Supporting Information).^[^ ^43^ ^]^ Based on the cooling and corresponding time‐ln*θ* curves (Figures S12 and S13, Supporting Information), the PCE (*η*) of **1P** was estimated as 67.58% (808 nm) and 61.36% (1064 nm), demonstrating competitive performance among NIR photothermal materials (Table S2, Supporting Information). To approach more realistic application conditions, we further investigated the photothermal conversion performance of **1P** pellet under Xe lamp irradiation (Figure S14, Supporting Information). As the light intensity increased from 0.5 Sun to 1 Sun, the temperature of **1P** pellet gradually rose, showing a clear power‐dependent photothermal response. Moreover, after ten irradiation on/off cycles, the sample maintained good stability, demonstrating its potential for practical applications under real solar illumination.

Given the high NIR‐PCE of **1P**, we further analyzed the NIR‐II photothermal process as a representative case. TD‐DFT calculations indicate that the broad NIR absorption (750–2500 nm) originates from π‐stacked Hox^•^–H_2_bV^•^ radical pairs. Additionally, fluorescence measurements under xenon lamp irradiation show a gradual decrease in emission intensity with prolonged exposure (Figure S15, Supporting Information), suggesting enhanced non‐radiative decay in **1P**. This behavior is attributed to competitive electron transfer processes, consistent with reports that such processes facilitate non‐radiative deactivation of excited states.^[^ ^41^ ^]^ Specifically, the strong *π* interactions facilitate close molecular packing and enhanced intermolecular electronic coupling, which promote efficient exciton delocalization, vibrational relaxation, and efficient light absorption.^[^ ^41^, ^42^ ^]^ These processes accelerate non‐radiative decay pathways, thereby converting absorbed photon energy into heat more effectively.^[^ ^44^ ^]^ Moreover, the face‐to‐face stacking configuration of the conjugated cores reduces radiative recombination and strengthens phonon–phonon interactions, further boosting photothermal conversion efficiency.^[^ ^45^ ^]^ Therefore, the efficient NIR‐II photothermal performance of **1P** is attributed to synergistic effects of electron transfer and extensive π‐stacking between Hox^•^ and H_2_bV^•^ radicals, which together promote efficient non‐radiative dissipation of photoexcited energy.

### 2.4 Photo‐Thermo‐Electric Conversion

**Figure 3:**
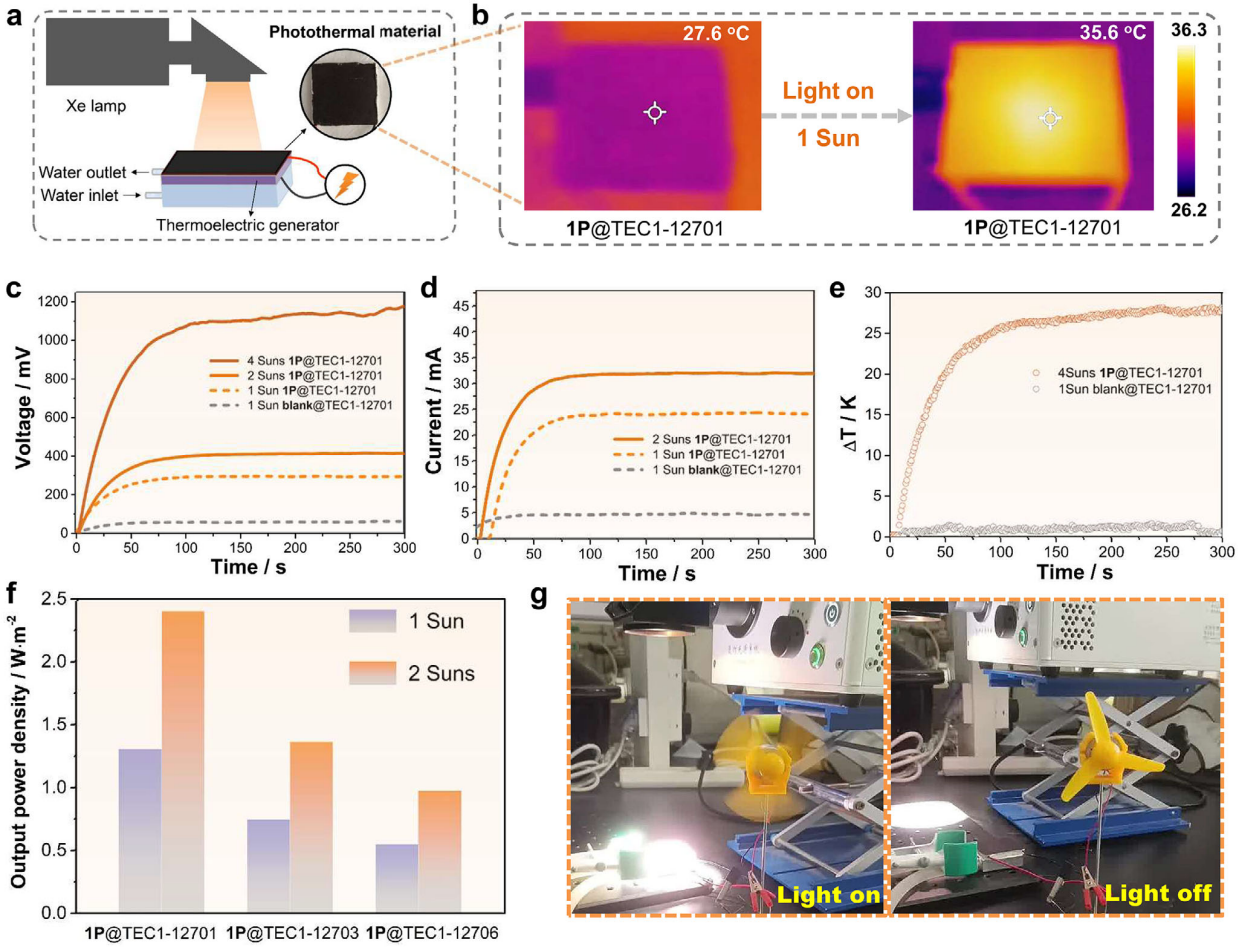
a) Schematic diagram of a photothermal‐electric conversion device. b) Infrared photos of the device before and after irradiation by Xe lamp. Open circuit voltages c) and currents d) of **1P**@TEC1‐12701 device under the irradiation of 1 Sun, 2 Suns or 4 Suns Xe lamp, respectively. e) The temperature difference (**Δ** *T*) (k) of **1P**@TEC1‐12701 device under the irradiation of 4 Suns Xe lamp. f) The output power density of different photo‐thermo‐electric conversion systems when loading external resistances under the irradiation of 1 Sun and 2 Suns Xe lamp. g) The pictures of the device driving a small fan (left: light on; right: light off).

Owing to its full solar spectrum absorption and efficient NIR photothermal effects, the photo‐thermo‐electric conversion application of **1P** was investigated. A 50 mg sample of **1P** crystalline powder was uniformly smeared onto the surface of a commercial thermoelectric generator (TEC1‐12701) using thermal conductive glue (**Figure** **3**a). Under 1 Sun illumination (1000 W·m^−2^, CEL‐PF300‐T9), the resulting device (**1P**@TEC1‐12701) generated an open‐circuit voltage of 292.3 mV (Figure 3c) and a current of 24.0 mA (Figure 3d). To assess the effect of device configuration, identical coatings of **1P** were applied to two other commercial modules (TEC1‐12703 and TEC1‐12706). Under the same illumination, **1P**@TEC1‐12703 produced 166.5 mV and 26.3 mA (Figure S16a,b, Supporting Information), while **1P**@TEC1‐12706 yielded 110.5 mV and 30.1 mA (Figure S16c,d, Supporting Information). Without **1P**, all modules exhibited negligible temperature changes (< 1 K), whereas coatings of **1P** significantly enhanced thermal gradients, confirming its strong photothermal effect (Figure S17, Supporting Information). Notably, **1P**@TEC1‐12701 showed the highest Δ*T* of 8 K (Figure 3b), underscoring the importance of interface quality in optimizing photo‐thermo‐electric performance. As expected, output voltage and current increased with light intensity.^[^ ^6^ ^]^ Under 2 Suns (2000 W·m^−2^), **1P**@TEC1‐12701 achieved an open‐circuit voltage of 412.8 mV and a current of 31.9 mA (Figure 3c,d; Figure S16, Supporting Information), further demonstrating its potential in practical solar energy harvesting.

To evaluate the maximum output power of different thermoelectric generators loaded with **1P** under 1 Sun and 2 Suns, a series of external resistances was applied. The optimal external resistance for highest power output was 9.3 Ω for **1P**@TEC1‐12701, 8.4 Ω for **1P**@TEC1‐12703, and 3.2 Ω for **1P**@TEC1‐12706 (Figure S18a–f, Supporting Information). Although **1P**@TEC1‐12701 did not generate the highest output current (Figure S19, Supporting Information), it achieved the highest power density, reaching 1.30 W·m^−2^ under 1 Sun and 2.40 W·m^−2^ under 2 Suns (Figure 3f). In comparison, under 1 Sun, **1P**@TEC1‐12703 and **1P**@TEC1‐12706 yielded power densities of 0.74 and 0.55 W·m^−2^, respectively; under 2 Suns, these increased to 1.36 and 0.97 W·m^−2^ (Figure 3f). These results confirm that **1P**@TEC1‐12701 performs superior photo‐thermo‐electric performance among the tested configurations.

To clarify the performance differences among the three thermoelectric modules (TEC1‐12701, TEC1‐12703, and TEC1‐12706), we obtained detailed specifications from the supplier, as presented in Table S4 (Supporting Information). The higher open‐circuit voltage observed for the TEC1‐12701 module relative to TEC1‐12703 and TEC1‐12706 can be attributed to differences in thermoelectric leg geometry and internal resistance.^[^ ^6^ ^]^ TEC1‐12701 features longer Bi_2_Te_3_‐based thermoelectric legs, which sustain a larger Δ*T* across each *p*–*n* junction. This increased Δ*T* enhances the effective Seebeck voltage produced by the 127 thermocouples connected in series. Additionally, the longer legs result in higher internal electrical resistance, which limits current under load but allows the open‐circuit voltage to approach its theoretical maximum. Together, these structural factors lead to the measured V_oc_ of TEC1‐12701 being higher under comparable thermal conditions.

To demonstrate practical applicability, a fan‐driving experiment was performed under intensified solar irradiation. Under 4 Suns, the open‐circuit voltage of the **1P**@TEC1‐12701 device reached 1.17 V (Figure 3c), with a temperature difference of up to 27 K (Figure 3e), sufficient to power a small fan (Figure 3g; Movie S1, Supporting Information). This performance exceeds that of most reported photothermal materials under comparable conditions (Table S1, Supporting Information), highlighting the real‐world potential of **1P** for solar energy harvesting. Given these results, even higher output is anticipated at greater light intensities or through serial device integration, further underscoring the promise of **1P** in advanced photo‐thermo‐electric technologies.

To assess the **1P**@TEC1‐12701 device stability under prolonged solar irradiation, additional experiments were carried out under 1 Sun illumination of a xenon lamp. During 26 h illumination, the device maintained a stable open‐circuit voltage of over 255 mV without noticeable degradation, demonstrating excellent operational stability and durability (Figure S20, Supporting Information). These findings indicate that both the material and the assembled device possess good long‐term stability under continuous solar exposure. To evaluate the practical applicability of the device, we have conducted additional outdoor experiments under natural sunlight. As shown in Figure S21 (Supporting Information), the open‐circuit voltage is consistent with the variation of solar irradiance during the day, which reaches the maximum output voltage of 216.9 mV (*I*_solar_ = 566 W/m^2^) at 10:31 in Liaocheng (China) on 3 November 2025. The performance is consistent with that obtained under simulated illumination, confirming the device's operational stability and practical feasibility.

## 3 Conclusion

In conclusion, we have demonstrated the first application of photochromism in photo‐thermo‐electric conversion, and developed a single‐component viologen‐based photochromic compound that serves as a full solar spectrum absorbing photothermal material. The compound exhibits excellent photochromic behavior, undergoing a reversible color change from yellow to black upon irradiation. The colored state displays strong absorption across 250–2500 nm and achieves a high PCE exceeding 60% in both NIR‐I and NIR‐II regions. Structural and theoretical analyses attribute these properties to the formation of stable π‐stacked radical interactions. When integrated into a thermoelectric device, the system generates an open‐circuit voltage of 292.3 mV and an output power density of 1.30 W·m^−2^ under 1 Sun, significantly outperforming conventional inorganic photothermal materials. Under intensified irradiation (4 Suns), the device can even drive a small fan, underscoring its practical potential. Therefore, this discovery provides a new strategy for efficient solar energy harvesting in photo‐thermo‐electric conversion technology.

## 4 Experimental Section

### Materials

All reagents were obtained from commercial sources and used without further purification. The ligand 1,1′‐bis(4‐carboxyphenyl)‐(4,4′‐bipyridinium) dichloride (H_2_bVCl_2_) was synthesized following a previously reported procedure.^[^ ^46^ ^]^

### Synthesis of Compound **1**

H_2_bVCl_2_ (0.1 mmol, 44 mg), oxalic acid dihydrate (0.1 mmol, 13 mg), and a solvent mixture of 2 mL distilled water and 2 mL acetonitrile were added to a 20 mL Teflon‐lined stainless‐steel autoclave, which was then sealed and heated at 100 °C for 2 days. Yellow flake‐like crystals of **1** were obtained by filtration (yield: ≈40% based on H_2_bVCl_2_). The phase purity of **1** was confirmed by powder X‐ray diffraction (PXRD) (Figure S1, Supporting Information) and elemental analysis. Anal. Calcd for 1 (M = 612.49 g mol^−1^): C, 54.91%; H, 3.95%; N, 4.57%. Found: C, 54.84%; H, 3.89%; N, 4.61%. Thermogravimetric (TG) and PXRD analyses revealed that **1** remains thermally stable up to at least 140 °C (Figure S2, Supporting Information).

### Measurements

Powder X‐ray diffraction (PXRD) patterns were collected at room temperature on a SmartLab 9 kW diffractometer using Cu Kα radiation (*λ* = 1.5406 Å, 450 W). Simulated PXRD patterns were generated using Mercury (Version 3.5.1), based on single‐crystal X‐ray diffraction data. Elemental analyses (C, H, and N) were performed using an Elementar Vario EL cube microanalyzer. Thermogravimetric (TG) analysis was conducted on a STA449F5‐QMS403D simultaneous thermal analyzer with Al_2_O_3_ crucibles under a nitrogen flow (20 mL min^−1^), at a heating rate of 10 K min^−1^ from 30 to 800 °C. Diffuse reflectance spectra were measured at room temperature over the 250–2500 nm range using a PerkinElmer Lambda 900 UV/vis/NIR spectrophotometer equipped with an integrating sphere. A BaSO_4_ plate was employed as the reference (100% reflectance), and finely ground sample powders were coated directly onto the reference plate. Electron paramagnetic resonance (EPR) spectra were recorded on a CIQTEK EPR‐200Plus spectrometer operating in the X‐band with a 100 kHz magnetic field modulation. A CEL‐PF300‐T9 xenon lamp was used as the light source for sample illumination during spectroscopic measurements.

### X‐Ray Crystallography

Single‐crystal X‐ray diffraction data for compound **1** were collected on a Bruker D8 QUEST diffractometer equipped with a graphite‐monochromated Mo Kα radiation source (*λ* = 0.71073 Å). The structure was solved and refined using the *Olex2* software suite.^[^ ^47^ ^]^ Atomic positions and displacement parameters were refined by full‐matrix least‐squares methods against F^2^. All non‐hydrogen atoms were refined anisotropically, while hydrogen atoms were placed in geometrically calculated positions and refined using a riding model. Crystallographic data for **1** are summarized in Table S3 (Supporting Information).

### The NIR Photothermal Conversion Measurement

NIR‐I/II photothermal conversion measurements were conducted by pressing pellets of **1P** with lasers of 808 and 1064 nm produced by Changchun New Industries Optoelectronics Tech. Co., Ltd, respectively. The temperature of the samples was recorded by a HIKMICRO K20 infrared camera. The temperature detecting range of the infrared camera was set as auto. A cylindrical tablet with a diameter of 5 mm and a weight of ≈15.5 mg was prepared using a tablet press.

### The Photo‐Thermo‐Electric Conversion Measurement

The photo‐thermo‐electric conversion of **1P** was investigated by integrating its crystalline powder with a commercial thermoelectric generator. Approximately 50 mg of **1P** powder was uniformly coated onto the surface of the thermoelectric chip using a thin layer of thermally conductive adhesive. Simulated solar irradiation at intensities of 1 Sun (1000 W m^−^^2^), 2 Suns (2000 W m^−2^), and 4 Suns (4000 W m^−2^) was provided by a xenon lamp (CEL‐PF300‐T9, CEAULIGHT) equipped with an attenuator, and the light intensity was measured using a TES1333 solar power meter. Three thermoelectric devices—TEC1‐12701 (40 × 40 × 5.2 mm^3^), TEC1‐12703 (40 × 40 × 4.4 mm^3^), and TEC1‐12706 (40 × 40 × 3.8 mm^3^)—served as thermoelectric generators (Table S4, Supporting Information). The open‐circuit voltage and current were measured with a Keithley 2450 source meter. Surface temperature variations during illumination were monitored using a HIKMICRO K20 infrared thermal camera.

### Calculation of Intermolecular Interactions

The electron density (ρ) and the corresponding reduced density gradient (s = 1/[2(3π^21^)^1\3^]·|∇ρ|/ρ^4\3^) were obtained via density functional theory (DFT) calculations. All calculations were performed using the B3LYP^[^ ^48^ ^]^ functional in conjunction with the 6–311G(d,p) basis set, as implemented in the Gaussian 16 software package.^[^ ^49^ ^]^

### Calculation of Optical Absorption Lines and Spin Density

Structural models of H_2_bV^•^, [2H_2_bV^•^], 2[H_2_bV^•^], [(Hox^•^)(H_2_bV^•^)], 2[(Hox^•^)(H_2_bV^•^)]‐a and 2[(Hox^•^)(H_2_bV^•^)]‐b were derived from the crystallographic data of compound **1**. Owing to the electron‐transfer absorption, calculations were carried out using the B3LYP functional with the 6–311G(d,p) basis set in Gaussian 16. The resulting wavefunction data were all analyzed using the Multiwfn program.^[^ ^50^ ^]^

### Calculation of Density of States (DOS)

The DOS and partial DOS (PDOS) calculations were carried out using the CASTEP^[^ ^51^ ^]^ module implemented in Materials Studio 8.0. The structural model of compound **1** was constructed directly based on single‐crystal X‐ray diffraction data. The exchange–correlation energy was treated using the Perdew–Burke–Ernzerhof (PBE) functional within the generalized gradient approximation (GGA).^[^ ^52^, ^53^ ^]^ Norm‐conserving pseudopotentials were employed to describe the electron–ion interactions.^[^ ^54^, ^55^ ^]^ A plane‐wave cutoff energy of 750 eV was applied. The Fermi level was defined as the reference energy and set to 0 eV. The smearing width was set to 0.05 eV. All other parameters were kept at their default values.

[CCDC 2475835 contains the supplementary crystallographic data for this paper. These data can be obtained free of charge from The Cambridge Crystallographic Data Centre via www.ccdc.cam.ac.uk/data_request/cif.]

## Conflict of Interest

The authors declare no conflict of interest.

## Supporting information



Supporting Information

Supporting Information

Supplemental Movie 1

## Data Availability

The data that support the findings of this study are available from the corresponding author upon reasonable request.
