# Supplementary material for: Photochromism‐Driven Full Solar Spectrum Absorption for Efficient Photo‐Thermo‐Electric Conversion
Source: Adv Sci (Weinh). 2025 Nov 27;13(8):e18602. doi: 10.1002/advs.202518602 (PMC12884802; doi:10.1002/advs.202518602)
Supplement: Supplementary file 1 — Supporting Information [file ADVS-13-e18602-s003.docx]

Supporting Information

Photochromism-Driven Full Solar Spectrum Absorption for Efficient Photo-Thermo-Electric Conversion

Ning-Ning Zhang,* Lin-Xu Liu, Zhen-Yu Li, Yong-Fang Han, Wen-Wen Zi, Kong-Gang Qu, Ming-Sheng Wang,* and Yong Yan*

**Table S1.** Reported photothermal materials and their performance in photo-thermo-electric conversion when integrating with thermoelectric generators under the irradiation of 1 Sun.

| Items | Photo-thermo-electric conversion (1 Sun) | | | References |
| --- | --- | --- | --- | --- |
|  | Open circuit voltage (mV) (TE module) | Temperature gradient (K) (TE module) | Maximum output power density (W/m^2^) (TE module) |  |
| **Organic photothermal materials** | | | | |
| TQC | **122.8** (TEC1−12706)  **427** (TEC1−12701) | **7.6** (TEC1−12706)  / (TEC1−12701) | **1.31** (TEC1−12706)  **2.21** (TEC1−12701) | *ACS Energy Lett.* **2023**, 8, 4179−4185. |
| [(H_2_bV)(Hox)_2_⋅2H_2_O] (**1P**) | **292.3** (TEC1−12701)  **166.5** (TEC1−12703)  **110.5** (TEC1−12706) | **8** (TEC1−12701)  **4** (TEC1−12703)  **4** (TEC1−12706) | **1.30** (TEC1−12701)  **0.74** (TEC1−12703)  **0.55** (TEC1−12706) | **This work** |
| CBC | **143** (TEG 1–127–3.6–3) | **18** (TEG 1–127–3.6–3) | N/A | *Natl Sci Rev* **2025**, 12, nwaf121. |
| 4OCSPC | **124** (TEC1−12706) | **4.6** (TEC1−12706) | N/A | *J. Mater. Chem. A.* **2021**, 9, 24452–24459. |
| 2TP-BBT | **95.5** (TEC1−12706) | **3.8** (TEC1−12706) | N/A | *J. Mater. Chem. A* **2023**, 11, 15380–15388. |
| DDPA-PDN | **83** (TEC1−12706) | **2.7** (TEC1−12706) | N/A | *Adv. Funct. Mater.* **2021**, 2106247. |
| GDPA-QCN | **56** (TEC1−12706) | **5** (TEC1−12706) | N/A | *Angew. Chem. Int. Ed.* **2022**, e202117087. |
| **Organic-inorganic hybrid photothermal materials** | | | | |
| {[BaMn(ONDI)_2_(H_2_O)_3_]⋅H_2_O}_n_ | **313** (TEC1−12701)  **161** (TEC1−12703)  **95** (TEC1−12706) | **7** (TEC1−12701)  **4.5** (TEC1−12703)  **4** (TEC1−12706) | N/A | *Chem. Eng. J.* **2024**, 491, 152054. |
| {[Ni_4_Cl_2_(ONDI)_2_(bpy)_4_]⋅2Cl⋅2H_2_O⋅*x*DMF⋅*y*H_2_O}_n_ | **250** (TEC1−12701)  **166.5** (TEC1−12703)  **110.5** (TEC1−12706) | **7** (TEC1−12701)  **6** (TEC1−12703)  **6** (TEC1−12706) | **0.53** (TEC1−12701)  **0.35** (TEC1−12703)  **0.26** (TEC1−12706) | *Chem. Eng. J.* **2024**, 499, 156059. |
| **Inorganic photothermal materials** | | | | |
| PCC-800 | **201** (SP1848) | **9.5** (SP1848) | **0.8** (SP1848) | *ACS Sustainable Chem. Eng*. **2022**, 10, 16427−16439 |
| MnO/C-600 | **177** (SP1848-27145) | **2** (SP1848-27145) | **0.77** (SP1848-27145) | *Chem. Eng. J.* **2023**, 451, 138534. |
| MC10 | **168.3** (SP1848-27145) | **7.9** (SP1848-27145) | **0.6** (SP1848-27145) | *Energy Environ. Mater.* **2023**, 6, e12376. |
| PC-x hydrogels | **165.8** (SP1848-27145) | **22.2** (SP1848-27145) | **0.65** (SP1848-27145) | *Chem. Eng. J.* **2023**, 458, 141511. |
| MCS1 | **140.6** (SP1848-27145) | **23.4** (SP1848-27145) | **0.42** (SP1848-27145) | *Adv. Energy Mater.* **2023**, 13, 2302451. |
| CNP | **115** (Al_2_O_3_ ceramic) | **5** (Al_2_O_3_ ceramic) | **5** × **10^–5^** (Al_2_O_3_ ceramic) | *Nano Energy* **2018**, 48, 481–488. |
| MSx-CPC | **110** (TEC1-12706) | **18** (TEC1-12706) | **1.087** (TEC1-12706) | *Small* **2022**, 18, 2201949. |
| CNTP | **100** (SP1848-27145) | N/A | **0.24** (SP1848-27145) | *Energy Convers. Manag.* **2021**, 241, 114306. |
| MoS_2_-x NSAs | **98.2** (F40550) | **17** (F40550) | **0.749** (F40550) | *Energy Convers. Manag.* **2022**, 252, 115070. |
| CNT foam/PVA | **96.35** (TEG1-12708-3.6) | **4.1** (TEG1-12708-3.6) | **0.4** (TEG1-12708-3.6) | *ACS Appl. Nano Mater.* **2021**, 4, 8906−8912. |
| PCC sponge | **60** (TEP1-097T200) | **4.6** (TEP1-097T200) | **0.4** (TEP1-097T200) | *Adv. Energy Mater.* **2019**, 1900250 |
| NF@RGO-CNT | **60** (TES1-12704) | N/A | **0.251** (TES1-12704) | *Ind. Eng. Chem. Res.* **2022**, 61, 16565−16576. |
| 3D porous CPP | **19.76** (TEP1-097T200) | **6** (TEP1-097T200) | **0.5** (TEP1-097T200) | *ACS Sustain. Chem. Eng.* **2021**, *9*, 4571−4582. |
| **Polymer photothermal materials** | | | | |
| CP@PVA | **112.9** (TG module containing Bi_2_Te_3_) | **20.2** (TG module containing Bi_2_Te_3_) | **1.04** (TG module containing Bi_2_Te_3_) | *Sci. China Mater.* **2022**, 65, 2491–2501. |
| PNPG/MoS_2_ | **110** (ET-127-10-13-RS) | **4** (ET-127-10-13-RS) | **0.23** (ET-127-10-13-RS) | *ACS Appl. Mater. Interfaces.* **2022**, 14, 1034−1044. |

**Table S2.** Reported photothermal materials and their photothermal conversion efficiency (*η*) under the irradiation of a 1064 nm laser**.**

| **Items** | ${}_{1064}$ | **Reference** |
| --- | --- | --- |
| **Organic Small molecules** | | |
| BAF4 | 80 % | *Angew. Chem. Int. Ed.,* **2021***,* 60*,* 22376–22384 |
| CY-1234 | 76.01 % | *Small,* **2023***,* 2307829 |
| P-Pc-HSA | 64.7 % | *RSC Adv.*, **2020**, 10, 22656–22662 |
| [(H_2_bV)(Hox)_2_⋅2H_2_O] (**1P**) | **63.82 %** | **This work** |
| **2** | 62.26 % | *Angew. Chem. Int. Ed.,* **2024**, e202400913 |
| Zn_4_-H_2_Pc/DP | 58.3 % | *Chem. Sci.*, **2019**, 10, 8246–8252 |
| DAF-OH_2_⊂GBox-4^4+^ | 47.4 % | *Angew. Chem. Int. Ed.,* **2023**, 135, e202301267 |
| DAF_2_⊂GBox-4^4+^ | 37.6% |  |
| TTF_2_⊂GBox-4^4+^ | 39.9% |  |
| CSM_2_ | 31.6 % | *Mater. Horiz.,* **2020**, 7, 1379–1386 |
| **COFs** | | |
| CNPs | 50.6 % | *Chem. Commun.,* **2020**, 56, 7793−7796 |
| Py-BPy-COF NPs | 55.2 % | *J. Am. Chem. Soc.,* **2019**, 141, 14433–14442 |
| **HOFs** | | |
| TQC@PFC-1 | 32 % | *J. Mater. Chem. B,* **2023**, 11, 8649−8656 |
| **Complexes** | | |
| [Sr(BCA)_2_(H_2_O)_2_]_n_ | 84.5 % | *Inorg. Chem. Front.,* **2024***,* 11, 4867–4875 |
| Cu-THQNPs | 51.34% | *ACS Appl. Mater. Interfaces,* **2018**, 10, 25203–25212 |
| Au@MOF | 48.5% | *Nano Lett.,* **2019**, 19, 6772–6780 |
| rPMo·cTMB | 48.4 % | *Adv. Healthcare Mater.,* **2022**, 11, 2102352 |
| pMOF-a | 32.2 % | *Chem. Commun.*, **2022**, 58, 11095–11098 |
| THPTS-Pb | 15.2 % | *Inorg. Chem.,* **2024***,* 63, 3327–3334 |
| **Polymers** | | |
| **Y1** | 67.9 % | *Chem. Sci.,* **2021**, 12, 5177–5184 |
| **Y2** | 69.5 % |  |
| **Y3** | 76.5% |  |
| PBBTDTS | 65.0 % | *Chem. Commun.*, **2020**, 56, 1093–1096 |
| SPNs3 | 60.0 % | *Chem. Commun.*, **2019**, 55, 9487–9490 |
| HPW@PANI | 57.76 % | *Int. J. Nanomed.*, **2022**, 17, 5565–5579 |
| 2MPT^2+•^CB | 54.6 % | *Angew. Chem., Int. Ed.*, **2019**, 58, 15526–15531 |
| N1@2P | 53.8 % | *Small,* **2023**, 19, 2300203 |
| P_3_ | 46.0 % | *ACS Nano*, **2019**, 13, 7345–7354 |
| NP^PSP-P^t | 43.2 % | *Adv. Mater.,* **2023**, 35, 2300048 |
| 2NDTA | 35 % | *Adv. Funct. Mater.,* **2024**, 2401627 |
| SPNs | 21.2 % | *ACS Appl. Mater. Interfaces,* **2020**, 12, 33492–33499. |
| SP1 | 2.3 % | *Angew. Chem.Int. Ed.,* **2023**, 62, e202301617 |
| SP2 | 46.4 % |  |
| SP3 | 44.9 % |  |
| SP4 | 46.5 % |  |
| SP5 | 42.4 % |  |
| **Inorganic materials** | | |
| N-Doping CDs | 81.3 % | *Carbon*, **2020**, 162, 220–233 |
| AuPBs | 80.8 % | *ACS Nano*, **2018**, 12, 2643–2651 |
| Au_3_Cu nanocrystals | 75.2 % | *Nanoscale Horiz.*, **2018**, 3, 624–631 |
| MoO_2_ NPs | 55.6 % | *Sci. China Mater.*, **2020**, 63, 1085–1098 |
| CS–RuO_2_ NPs | 52.5 % | *Chem. Commun.*, **2020**, 56, 3019–3022 |
| Pd Ncap | 49.2 % | *ACS Appl. Mater. Interfaces*, **2023**, 15, 39081–39098 |
| Sb-Doped SnO_2_ | 48.3 % | *Nanoscale*, **2018**, 10, 2542–2554 |
| Ni_9_S_8_ | 46.0 % | *Nanoscale*, **2019**, 11, 20161–20170 |
| Nb_2_C (MXene) | 45.6 % | *J. Am. Chem. Soc.*, **2017**, 139, 16235–16247 |
| V_2_C | 45.0 % | *ACS Nano*, **2019**, 13, 1499–1510 |
| TeO_2_/(NH_4_)_x_WO_3_ nanoribbons | 43.6 % | *Nano Lett.*, **2019**, 19, 1179–1189 |
| NIR-II-CD/BP hybrids | 28.4 % | *ACS Appl. Mater. Interfaces*, **2019**, 11, 44949–44960 |
| Si–Au | 24.1 % | *J. Mater. Chem. B*, **2019**, 7, 4393–4401 |
| EGaIn @SiO_2_-RGD | 22.43 % | *Nano Lett.*, **2019**, 19, 2128–2137 |
| SnSe–PVP nanorods | 20.3 % | *Mater. Horiz.*, **2018**, 5, 946–952 |
| Au NSs | 13.0 % | *J. Mater. Chem. B*, **2019**, 7, 2001–2008 |

**Table S3.** Crystal data and structural refinements for **1**.

|  | Compound 1 |
| --- | --- |
| **CCDC** | **2475835** |
| **Formula** | **C_28_H_24_N_2_O_14_** |
| **M_r_** | **612.49** |
| **Crystal size (mm^3^)** | **0.3*0.2*0.1** |
| **Crystal system** | **triclinic** |
| **Space group** | ***P***$\bar{\boldsymbol{1}}$ |
| ***a* (Å)** | **6.72570(10)** |
| ***b* (Å)** | **6.83300(10)** |
| ***c* (Å)** | **15.3044(3)** |
| ***α* (deg)** | **78.1410(10)** |
| ***β* (deg)** | **85.8100(10)** |
| ***γ* (deg)** | **90.0040(10)** |
| ***V* (Å^3^)** | **686.41(2)** |
| ***D*_calcd_ (g/cm^3^)** | **1.482** |
| ***Z*** | **1** |
| ***F*(000)** | **318.0** |
| **Abs coeff (mm^–1^)** | **0.121** |
| ***R*_1_^a^** | **0.0468(3086)** |
| ***ωR*_2_^b^** | **0.1258(3853)** |
| **GOF on *F*^2^** | **1.028** |

*^a^R*_1_ = ∑||*F*_o_|–|*F*_c_||/∑|*F*_o_|; *^b^ωR*_2_ = {∑*ω*[(*F*_o_)^2^–(*F*_c_)^2^]^2^/∑*ω*[(*F*_o_)_2_]^2^}^1/2^.

**Table S4.** Parameter list of thermoelectric modules.

| Model No. | *I_max_* (A) | *α*_eff (μV/K per couple) (μV/K per couple) | R (Ω) | Size (W×L×H) mm |
| --- | --- | --- | --- | --- |
| **TEC1-12701** | 1.3 | 157.5 | 6.3 | 40 × 40 × 5.2 |
| **TEC1-12703** | 3.9 | 144.4 | 3.8 | 40 × 40 × 4.4 |
| **TEC1-12706** | 6.0 | 131.2 | 2.0 | 40 × 40 × 3.8 |


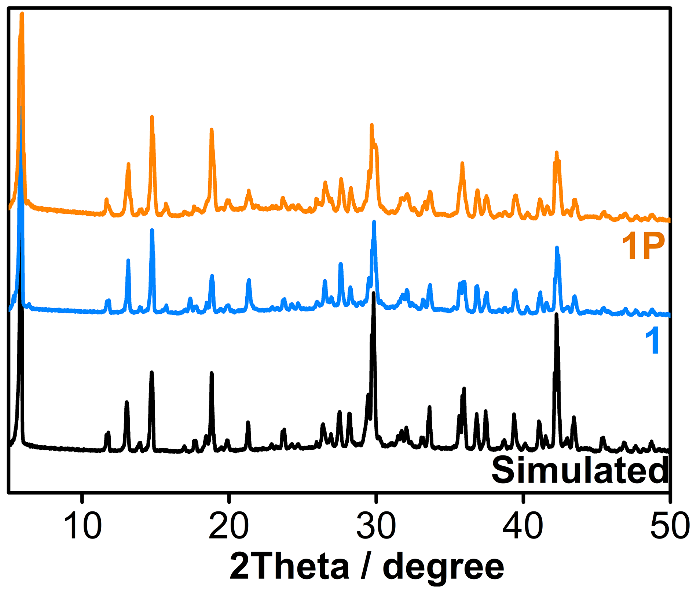


**Figure S1** PXRD patterns of **1**, **1P**, and the simulated data using single-crystal data.

**
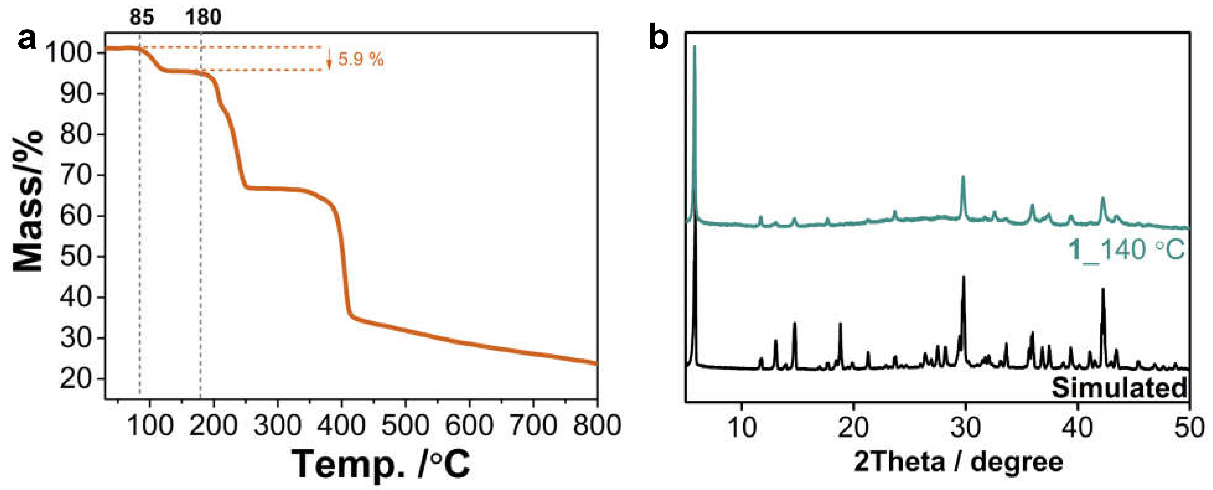
**

**Figure S2** (a) Thermogravimetric (TG) curve of **1**. (The initial mass loss of 5.9% corresponds to the release of lattice water molecules of **1**. Anal. Calcd for **1** (M = 612.49 g/mol): H_2_O, 5.88%). (b) PXRD patterns of **1** after annealing at 140 ^o^C for 2 hours, and the simulated data using single-crystal data. Note: The crystalline phase of **1** is retained up to at least 140  ^o^C, demonstrating its thermal stability.


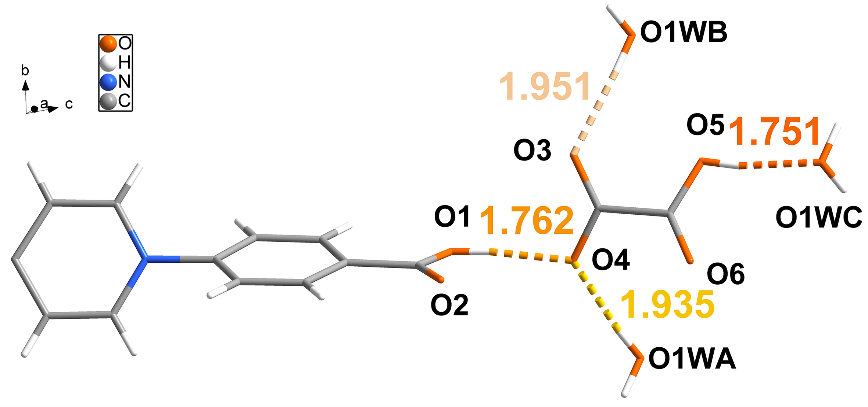


**Figure S3** A fragment of the crystal structure of **1**, showing four types of hydrogen bonds highlighted in different colours. Asymmetric unit code: A (2-x, -y, 2-z), B (2-x, 1-y, 2-z).


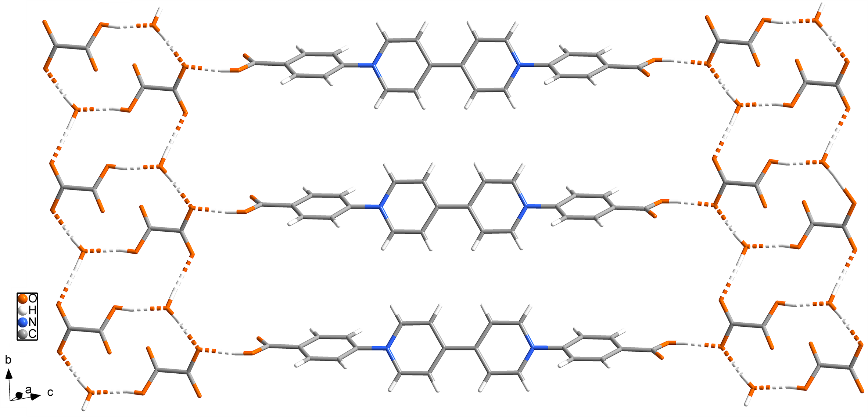


**Figure S4** Representation of the two-dimensional hydrogen-bonded framework in **1.**


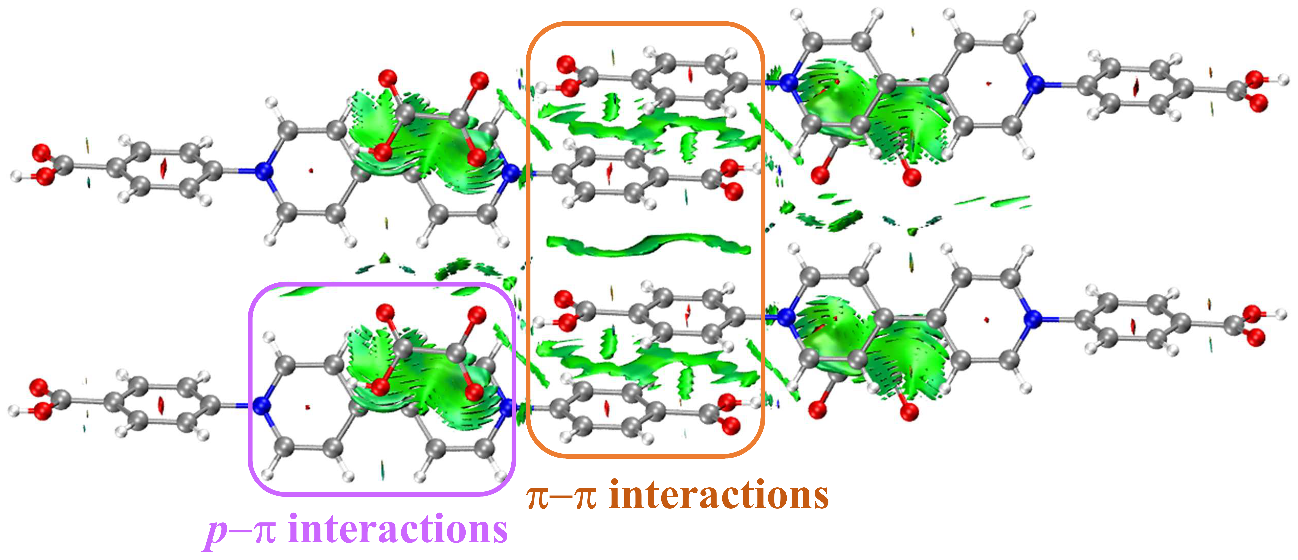


**Figure S5** A selected π-stacking fragment in **1** and its calculated gradient isosurfaces (s = 0.5 a.u.). The surfaces are coloured on a blue-green-red (BGR) scale according to values of sign(λ_2_)ρ, ranging from –0.04 to 0.02 a.u.. Blue, green, and red indicate strong attractive interactions, moderate attractive interactions, and strong non-bonded overlaps, respectively.


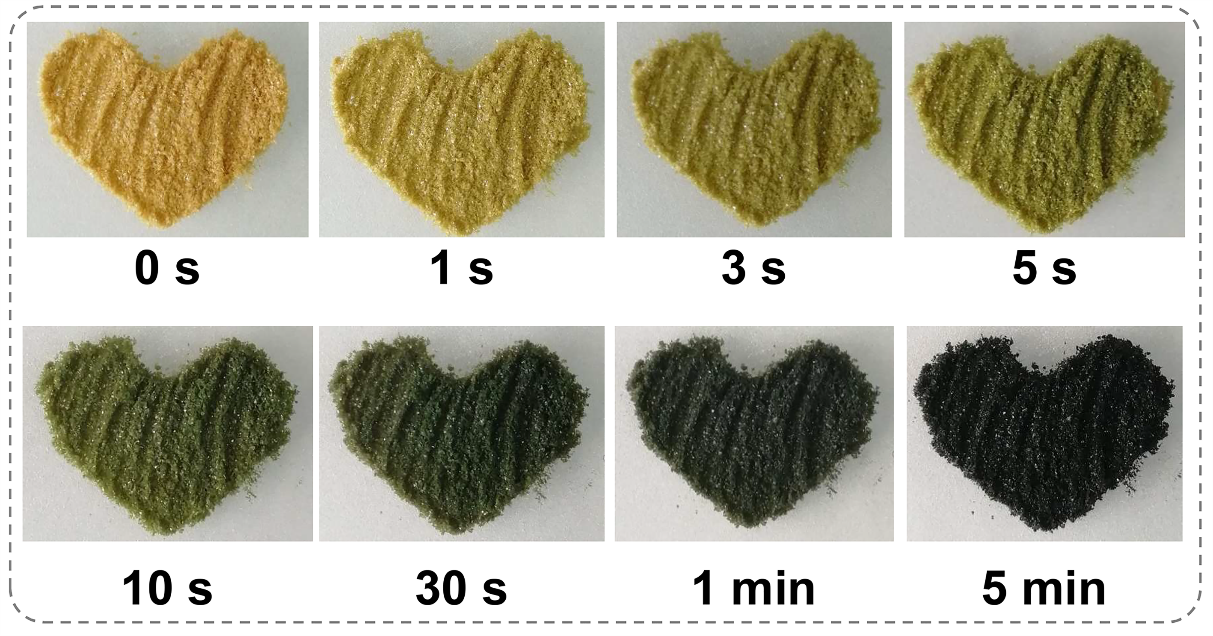


**Figure S6** Time-dependent color change of **1** upon irradiation of Xe lamp.


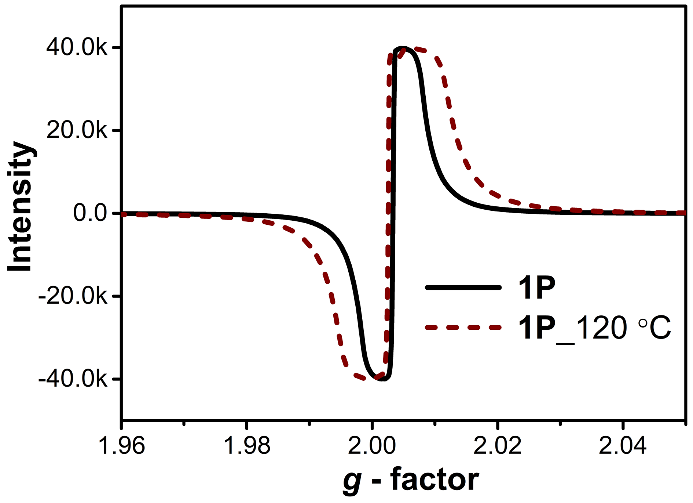


**Figure S7** Solid-state EPR spectra of **1P** (black solid line) and the sample **1P** after annealing at 120 °C for 2 hours (red dashed line).


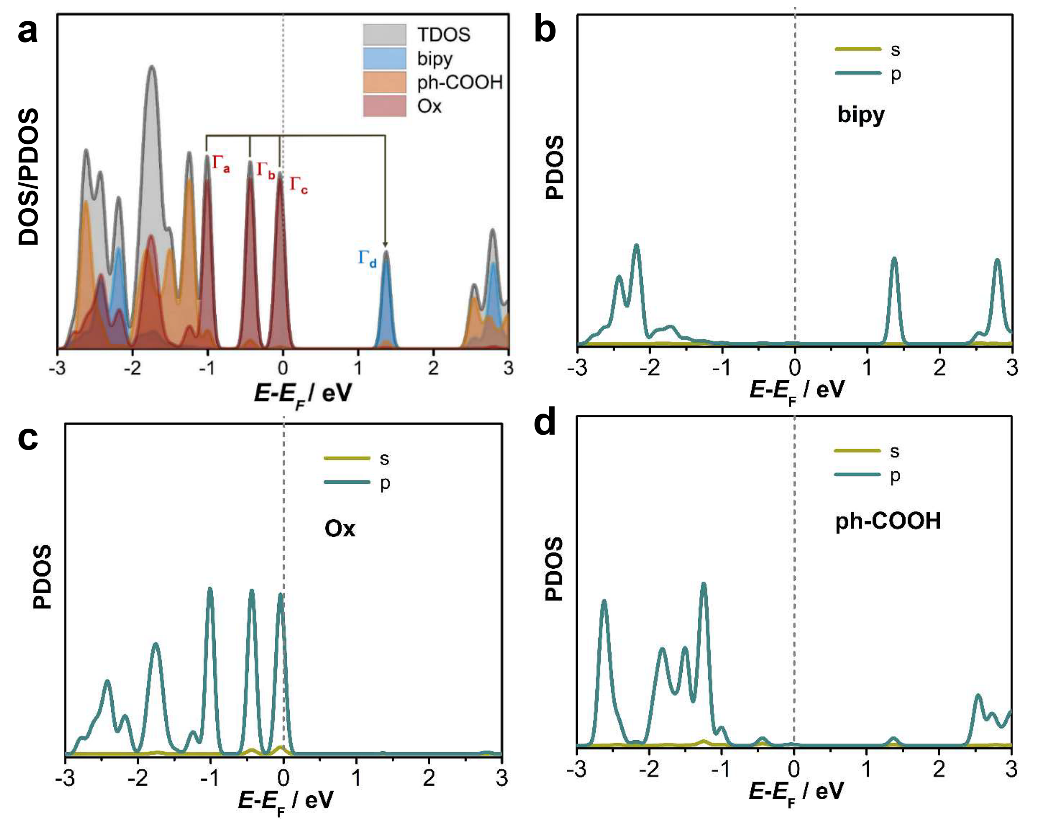


**Figure S8** (a) TDOS and PDOS of **1**. Partial DOS of bipy (b), Ox (c) and ph-COOH (d) in compound **1**. Note: Fermi level (E_F_) is conventionally set to zero.

**
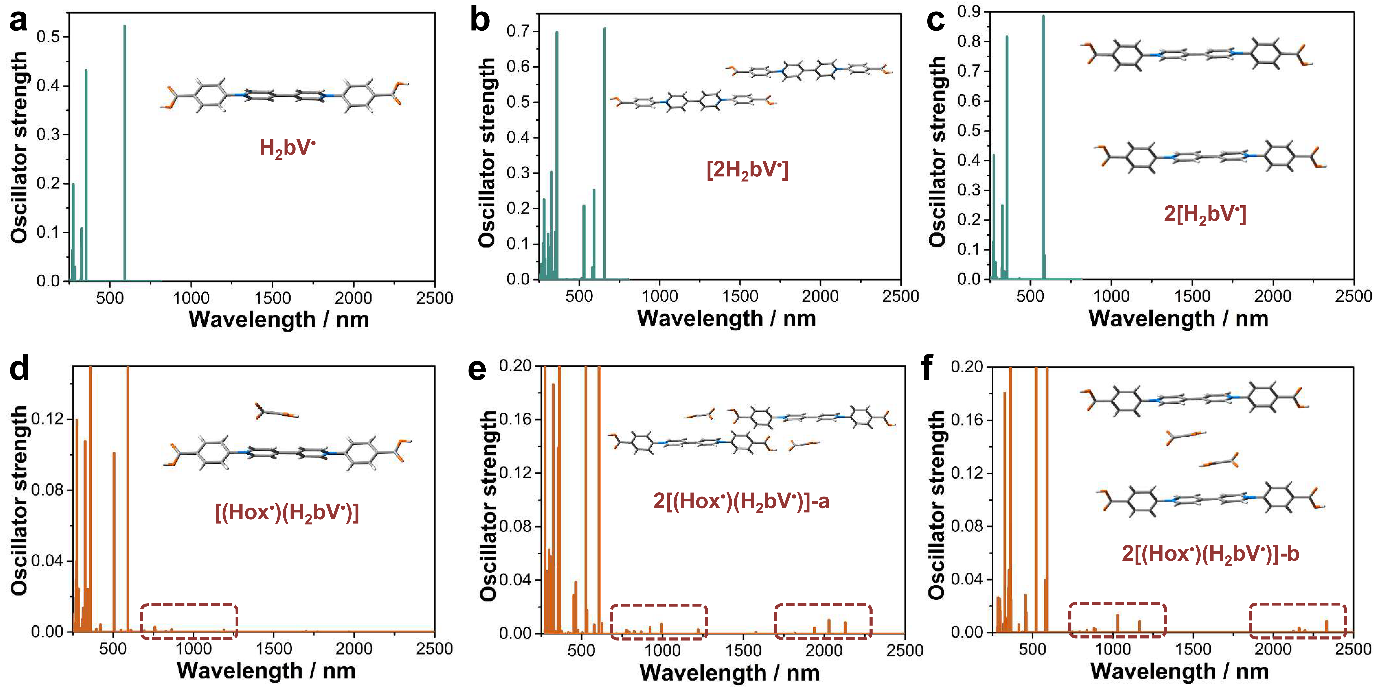
**

**Figure S9** (a) Optical absorption lines of the selected H_2_bV^•^ (**a**), [2H_2_bV^•^] (**b**), 2[H_2_bV^•^] (**c**), [(Hox^•^)(H_2_bV^•^)] (**d**), 2[(Hox^•^)(H_2_bV^•^)]-a (**e**), and 2[(Hox^•^)(H_2_bV^•^)]-b (**f**) units from the crystal structure, respectively.


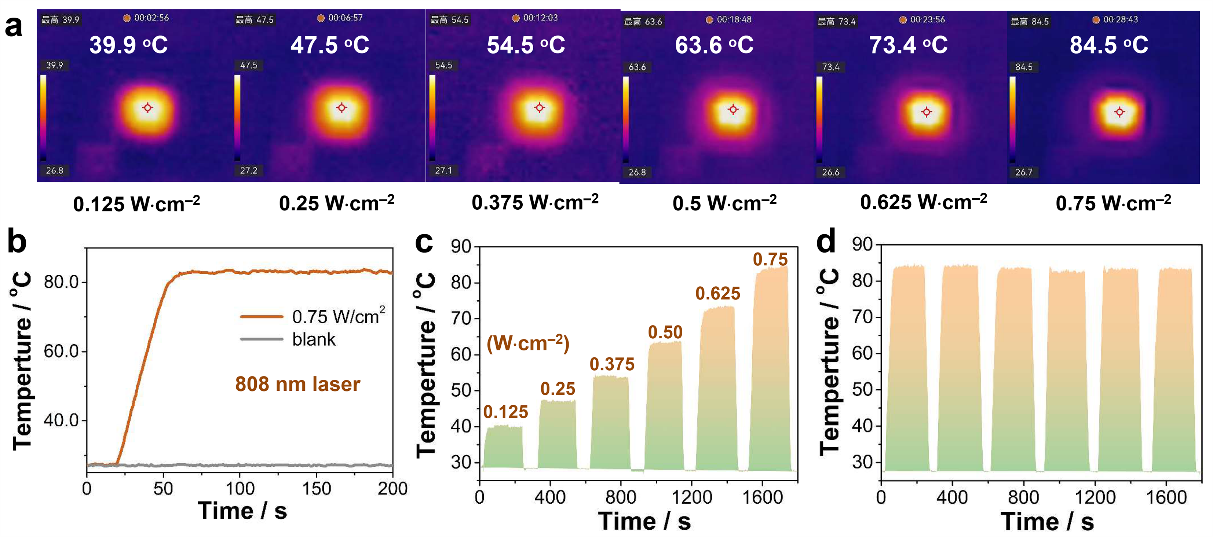


**Figure S10** (a) Infrared photos of crystalline pellet of **1P** under different irradiation power density. (b) Temperature curves of **1P** and blank quartz glass plate under the irradiation of 0.75 W/cm^2^ 808 nm laser. (c) Temperature changes of compound **1P** under the irradiation of 808 nm laser with different power density. (d) Cycling temperature curve of compound **1P** under the irradiation of 808 nm laser with a specific power densityof 0.75 W/cm^2^.


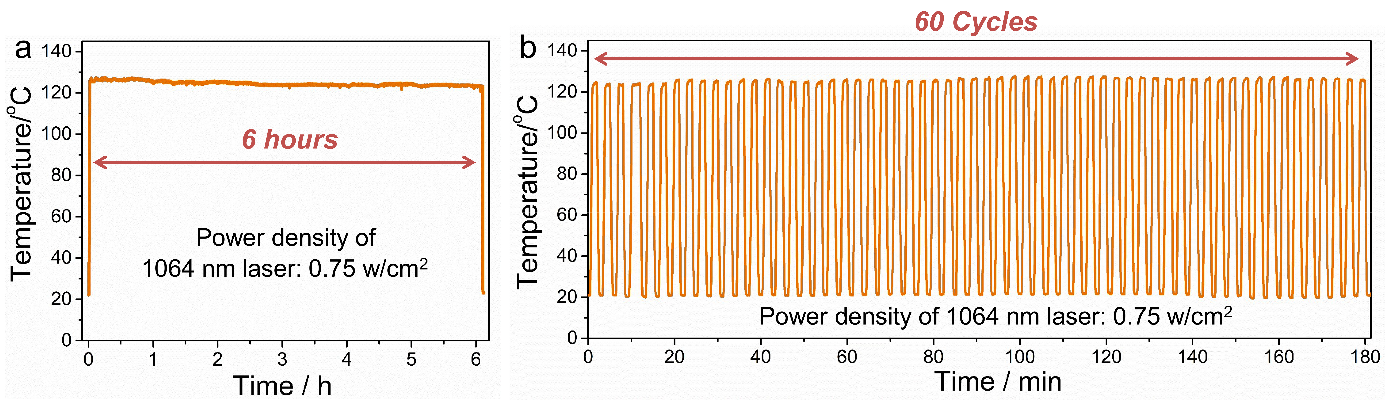


**Figure S11** (a) Temperature variation of the **1P** pellet under continuous 1064 nm laser irradiation (0.75 W cm⁻^2^) for 6 hours. (b) Photothermal stability of the **1P** pellet evaluated by 60 heating–cooling cycles under 1064 nm laser irradiation (0.75 W cm⁻^2^).


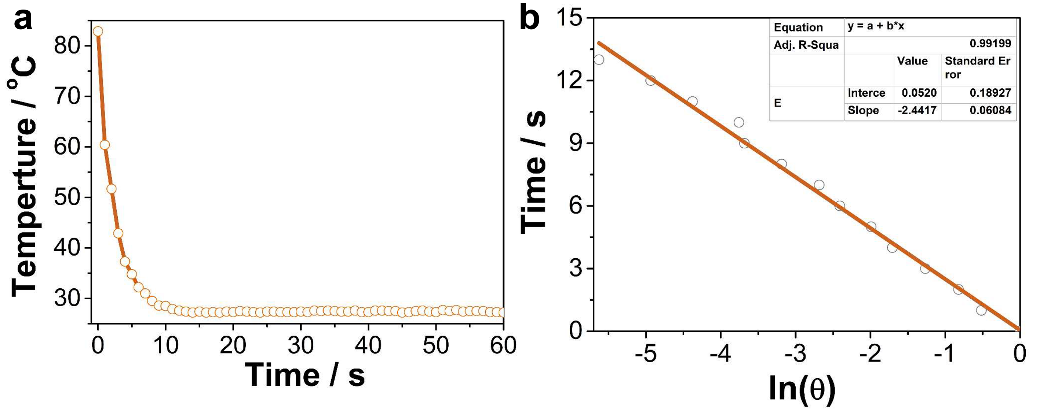


**Figure S12** Temperature decaying curve of **1P** after removing the laser source of 808 nm (0.75 W/cm^2^) (a) and the corresponding time-lnθ linear curve (b). The photothermal conversion efficiency (η_808_ = 69.46 %) was also calculated based on reported method^[^[[1]](#endnote-2)^]^: *η*_808_ = $\frac{\boldsymbol{hS}\boldsymbol{T}_{\boldsymbol{max}}}{\boldsymbol{I(1-}\boldsymbol{10}^{\boldsymbol{-}\boldsymbol{A}_{\boldsymbol{808}}}\boldsymbol{)}}$, where the *I* is the laser power (0.75 W/cm^2^), *A_808_* is the absorbance of the samples at the wavelength of 808 nm (0.69, F(R)), and ΔT_max_ is the maximum temperature change (57.8 K). *hs* can be calculated based on the formula of ${}_{\boldsymbol{s}}$ = $\frac{\sum_{\boldsymbol{i}} \boldsymbol{m}_{\boldsymbol{i}}\boldsymbol{C}_{\boldsymbol{p,i}}}{\boldsymbol{hs}}$, where ${}_{\boldsymbol{s}}$ is the sample system time constant, $\boldsymbol{m}_{\boldsymbol{i}}$ (0.0155 g) and $\mathbf{C}_{\boldsymbol{p,i}}$ (1.13 J⋅(g⋅^o^C)^−1^) are the mass and heat capacity of system components. When the laser turns off, ${}_{\boldsymbol{s}}$ can be estimated according to the formula: t = −${}_{\boldsymbol{s}}\boldsymbol{ln}$. The can be obtained according to the formula: = $\frac{\boldsymbol{T-}\boldsymbol{T}_{\boldsymbol{surr}}}{\boldsymbol{T}_{\boldsymbol{max}}\boldsymbol{-}\boldsymbol{T}_{\boldsymbol{surr}}}$, where T is the temperature of sample, T_max_ is the maximum system temperature, and T_surr_ is the environment temperature.


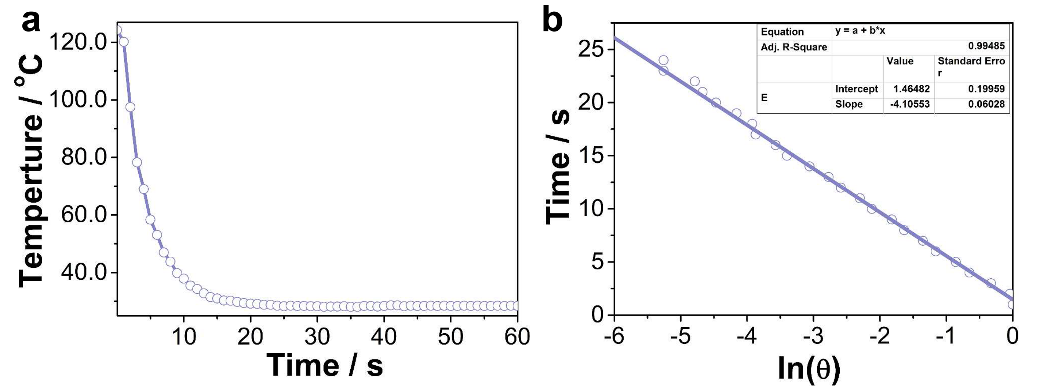


**Figure S13** Temperature decaying curve of **1P** after removing the laser source of 1064 nm (0.75 W/cm^2^) (a) and the corresponding time-lnθ linear curve (b). The photothermal conversion efficiency (η_1064_ = 63.82 %) was also calculated based on reported method^[11]^: *η*_1064_ = $\frac{\boldsymbol{hS}\boldsymbol{T}_{\boldsymbol{max}}}{\boldsymbol{I(1-}\boldsymbol{10}^{\boldsymbol{-}\boldsymbol{A}_{\boldsymbol{1064}}}\boldsymbol{)}}$, where the *I* is the laser power (0.75 W/cm^2^), *A_1064_* is the absorbance of the samples at the wavelength of 1064 nm (0.82, F(R)), and ΔT_max_ is the maximum temperature change (95.2 K). *hs* can be calculated based on the formula of ${}_{\boldsymbol{s}}$ = $\frac{\sum_{\boldsymbol{i}} \boldsymbol{m}_{\boldsymbol{i}}\boldsymbol{C}_{\boldsymbol{p,i}}}{\boldsymbol{hs}}$, where ${}_{\boldsymbol{s}}$ is the sample system time constant, $\boldsymbol{m}_{\boldsymbol{i}}$ (0.0155 g) and $\mathbf{C}_{\boldsymbol{p,i}}$ (1.13 J⋅(g⋅^o^C)^−1^) are the mass and heat capacity of system components. When the laser turns off, ${}_{\boldsymbol{s}}$ can be estimated according to the formula: t = −${}_{\boldsymbol{s}}\boldsymbol{ln}$. The can be obtained according to the formula: = $\frac{\boldsymbol{T-}\boldsymbol{T}_{\boldsymbol{surr}}}{\boldsymbol{T}_{\boldsymbol{max}}\boldsymbol{-}\boldsymbol{T}_{\boldsymbol{surr}}}$, where T is the temperature of sample, T_max_ is the maximum system temperature, and T_surr_ is the environment temperature.


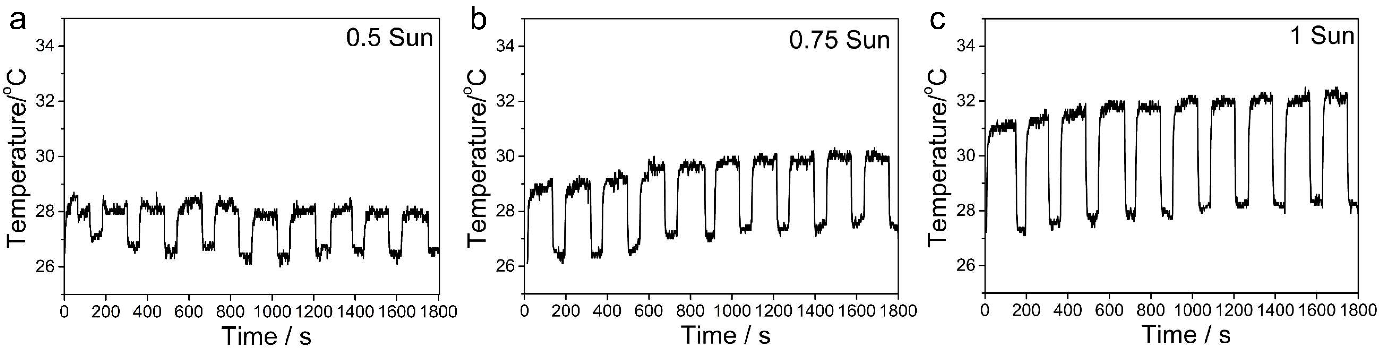


**Figure S14** 10 cyclic tests of the **1P** pellet (diameter: 5 mm; m = 17.5 mg) under different xenon lamp intensities of (a) 0.5, (b) 0.75, and (c) 1 Sun.


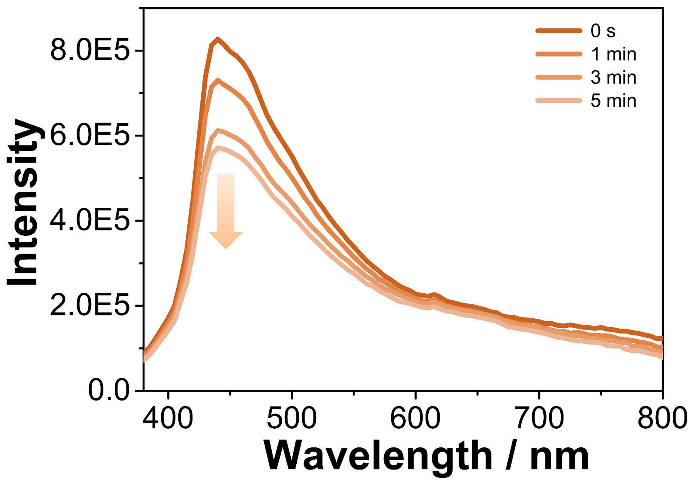


**Figure S15** Time-dependent solid-state photoluminescence spectra of **1** (λ_ex_ = 330 nm) at room temperature in air upon irradiation. It is obvious that the fluorescence of **1P** was decreased after photo irradiation, indicating the non-irradiative process in **1P** was enhanced by the photoinduced electron transfer process.


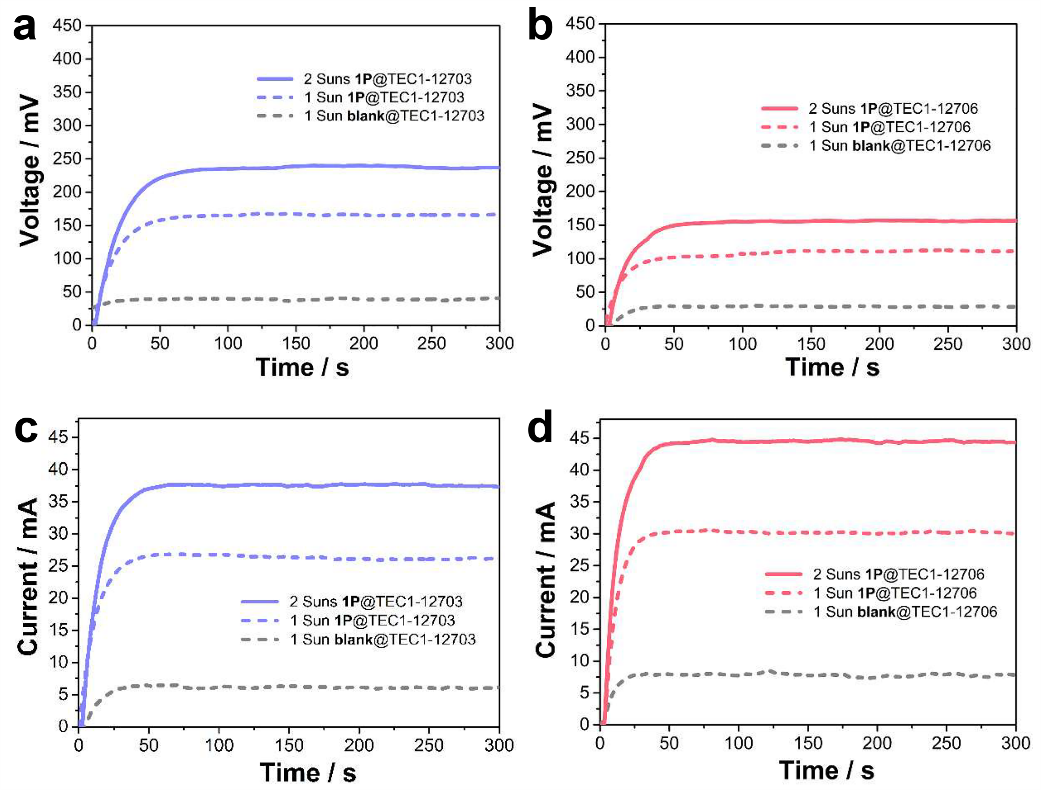


**Figure S16** Open circuit voltages (a, c) and currents (b, d) of **1P**@TEC1-12703 and **1P**@TEC1-12706 device under the irradiation of 1 Sun and 2 Suns Xe lamp, respectively.


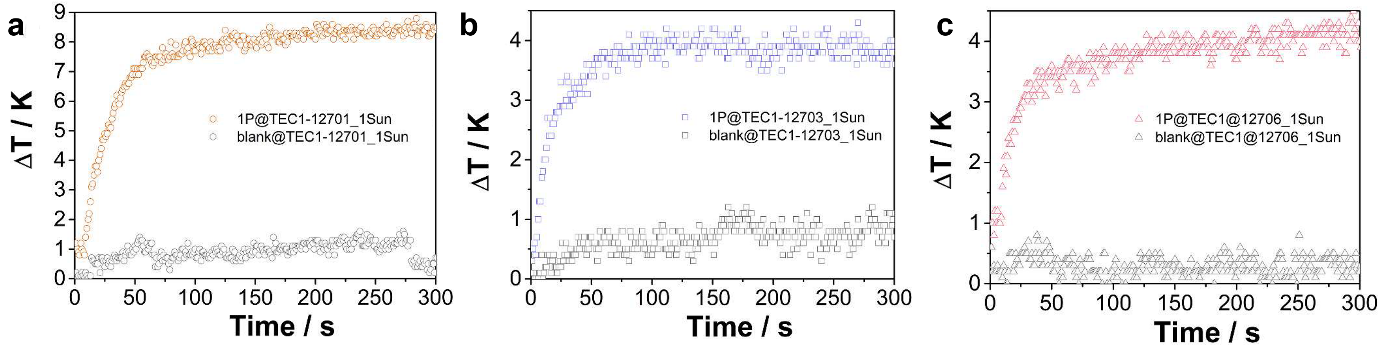


**Figure S17** The temperature difference (ΔT) of different photo-thermo-electric conversion devices under the irradiation of 1 Sun.


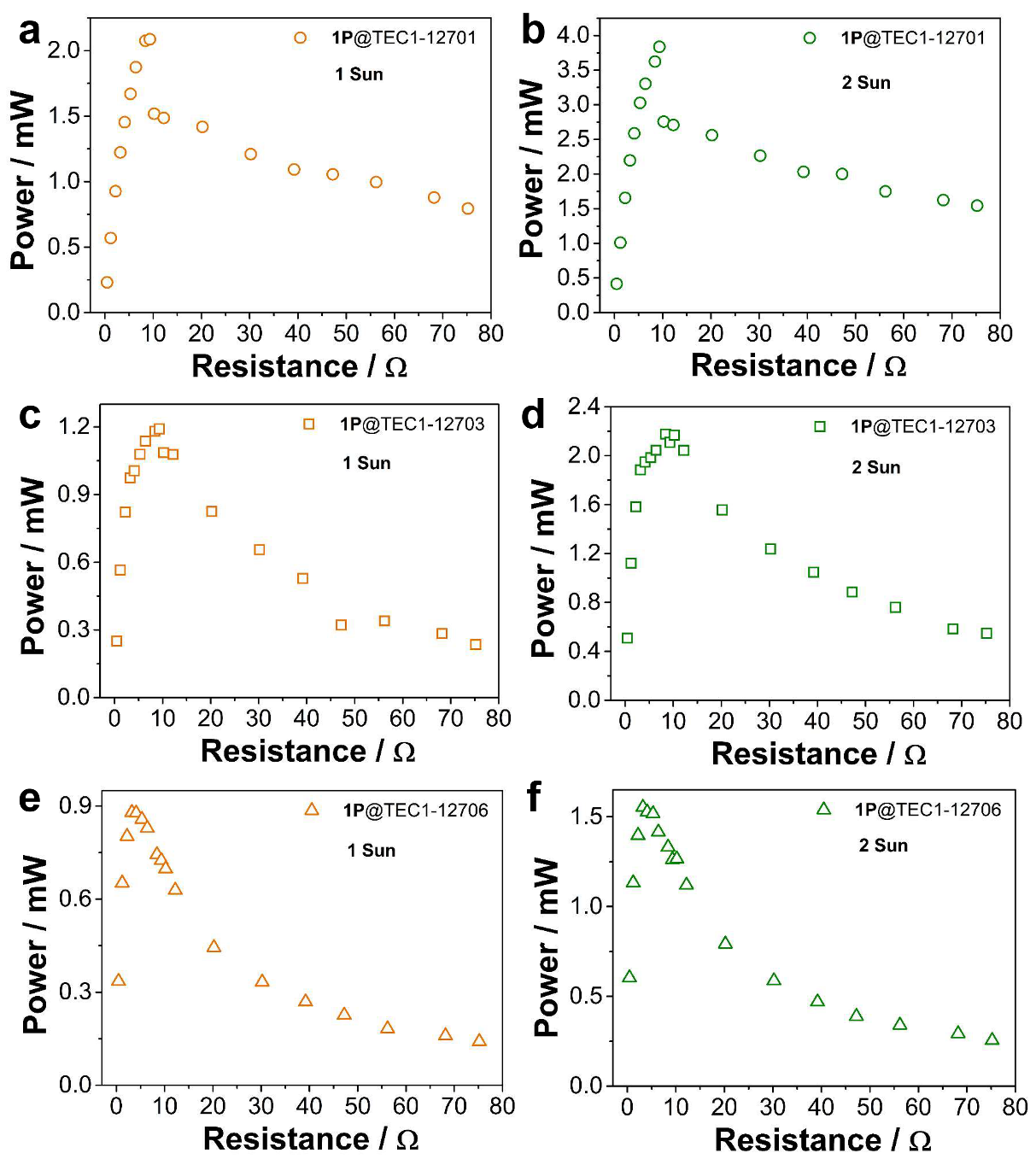


**Figure S18** Output power of **1P**@TEC1-12701 (a, b), **1P**@TEC1-12703 (c, d) and **1P**@TEC1-12706 (e, f) under irradiation of 1 Sun and 2 Sun when loading different external resistances, respectively.


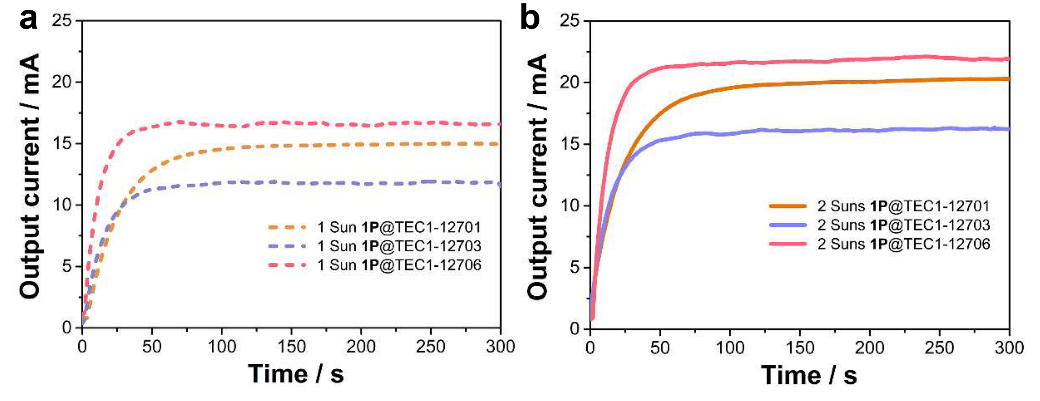


**Figure S19** The maximum output currrents of different photo-thermo-electric conversion systems when loading external resistances under the irradiation of 1 Sun (a) and 2 Suns (b).


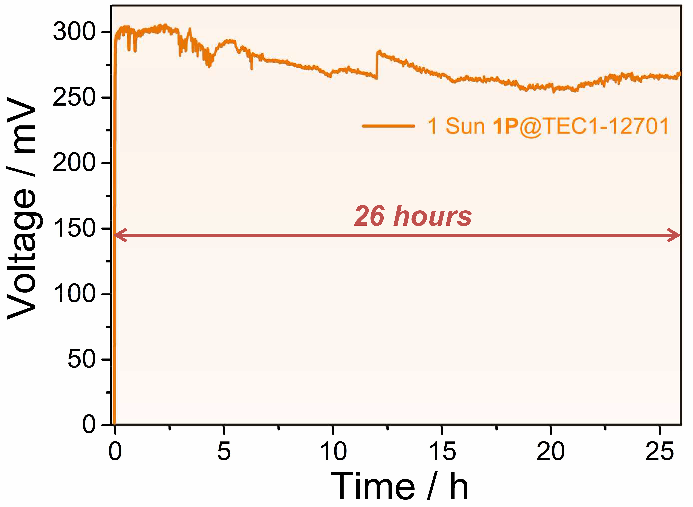


**Figure S20** The open-circuit voltage of the **1P**@TEC1-12701 device under 1 Sun illumination for 26 hours.


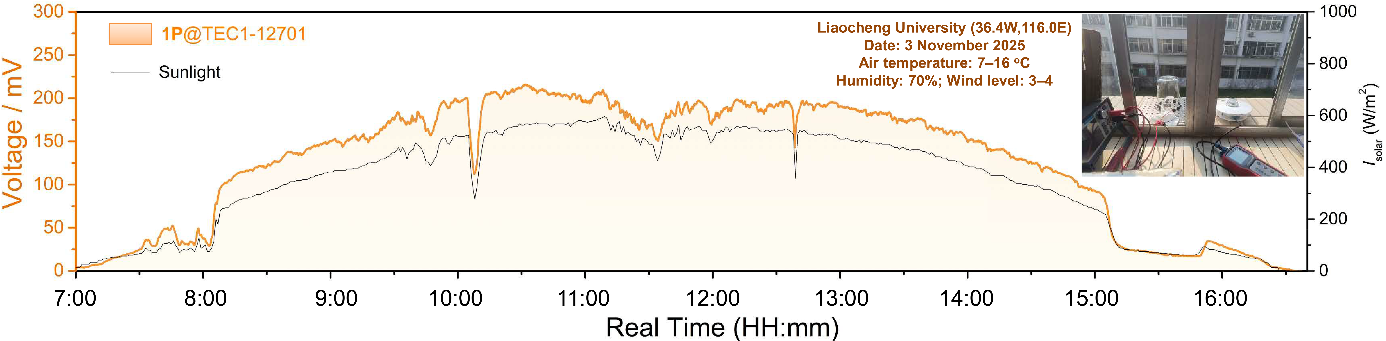


**Figure S21** The real-time open-circuit voltage data of the **1P**@TEC1-12701 device under natural light at Liaocheng (China) on 3 November 2025. Inset: Meteorological and topographic information of the experimental locations.

**Movie S1**

A thermoelectric device **1P**@TEC1-12701 drives a small fan under 4 Suns.

**References**

1. []. S. Wang, S. Li, J. Xiong, Z. Lin, W. Wei, Y. Xu, “Near-infrared photothermal conversion of stable radicals photoinduced from a viologen-based coordination polymer.” *Chem. Commun.* **2020**, 56, 7399–7402. [↑](#endnote-ref-2)
