# Supplementary material for: Photochromism‐Driven Full Solar Spectrum Absorption for Efficient Photo‐Thermo‐Electric Conversion
Source: Adv Sci (Weinh). 2025 Nov 27;13(8):e18602. doi: 10.1002/advs.202518602 (PMC12884802; doi:10.1002/advs.202518602)

checkCIF/PLATON report

Structure factors have been supplied for datablock(s) 1

THIS REPORT IS FOR GUIDANCE ONLY. IF USED AS PART OF A REVIEW PROCEDURE FOR PUBLICATION, IT SHOULD NOT REPLACE THE EXPERTISE OF AN EXPERIENCED CRYSTALLOGRAPHIC REFEREE.

No syntax errors found. CIF dictionary Interpreting this report

Datablock: 1

Bond precision:	C-C = 0.0018 A		Wavelength=0.71073
Cell:	a=6.7257(1)	b=6.8330(1)	c=15.3044(3)
	alpha=78.141(1)	beta=85.810(1)	gamma=90.004(1)
Temperature:	293 K		

	Calculated	Reported
Volume	686.41(2)	686.41(2)
Space group	P -1	P -1
Hall group	-P 1	-P 1
Moiety formula	C24 H18 N2 O4, 2(C2 H O4), 2(H2 O)	2(H2 O), 2(C2 H O4), C24 H18 N2 O4
Sum formula	C28 H24 N2 O14	C28 H24 N2 O14
Mr	612.49	612.49
Dx,g cm-3	1.482	1.482
Z	1	1
Mu (mm-1)	0.121	0.121
F000	318.0	318.0
F000'	318.21	
h,k,lmax	9,9,21	9,9,21
Nref	3868	3853
Tmin,Tmax	0.971,0.988	0.693,0.746
Tmin'	0.964	

Correction method= # Reported T Limits: Tmin=0.693 Tmax=0.746
AbsCorr = MULTI-SCAN

Data completeness= 0.996 Theta(max)= 29.587

R(reflections)= 0.0468(3086) wR2(reflections)= 0.1258(3853)

S = 1.028 Npar= 204

The following ALERTS were generated. Each ALERT has the format
test-name_ALERT_alert-type_alert-level.
Click on the hyperlinks for more details of the test.

Alert level C

```
PLAT042_ALERT_1_C Calc. and Reported MoietyFormula Strings  Differ     Please Check
              Calc: C24 H18 N2 O4, 2(C2 H O4), 2(H2 O)
              Rep.: 2(H2 O), 2(C2 H O4), C24 H18 N2 O4
PLAT094_ALERT_2_C Ratio of Maximum / Minimum Residual Density ....       2.09 Report
PLAT155_ALERT_4_C The Triclinic Unitcell is NOT Reduced .........     Please Do !
PLAT250_ALERT_2_C Large U3/U1 Ratio for <U(i,j)> Tensor(Resd    1)        2.6 Note
PLAT250_ALERT_2_C Large U3/U1 Ratio for <U(i,j)> Tensor(Resd    2)        2.3 Note
PLAT906_ALERT_3_C Large K Value in the Analysis of Variance ......      2.422 Check
PLAT911_ALERT_3_C Missing FCF Refl Between Thmin & STh/L=    0.600         13 Report
                1  0  0,  -2  1  0,   0  1  0,   1  1  0,   0  2  0,  -2  0  1,
               -1  0  1,  -2  1  1,  -1  1  1,  -1  2  1,   0  2  1,   2  0  2,
                0  2  2,
PLAT913_ALERT_3_C Missing # of Very Strong Reflections in FCF ....          9 Note
               -2  1  0,   1  1  0,   0  2  0,  -2  0  1,  -2  1  1,  -1  1  1,
               -1  2  1,   0  2  1,   0  2  2,
```

Alert level G

```
PLAT007_ALERT_5_G Number of Unrefined Donor-H Atoms .............           4 Report
              H1    H5    H1WA  H1WB
PLAT154_ALERT_1_G The s.u.'s on the Cell Angles are Equal ..(Note)      0.001 Degree
PLAT199_ALERT_1_G Reported _cell_measurement_temperature ..... (K)        293 Check
PLAT200_ALERT_1_G Reported   _diffrn_ambient_temperature ..... (K)        293 Check
PLAT720_ALERT_4_G Number of Unusual/Non-Standard Labels .........           2 Note
              H1WA    H1WB
PLAT790_ALERT_4_G Centre of Gravity not Within Unit Cell: Resd.  #          3 Note
              H2 O
PLAT910_ALERT_3_G Missing # of FCF Reflection(s) Below Theta(Min).          1 Note
                0  0  1,
PLAT912_ALERT_4_G Missing # of FCF Reflections Above STh/L=  0.600          1 Note
PLAT933_ALERT_2_G Number of HKL-OMIT Records in Embedded .res File          1 Note
               -1  1  1,
PLAT941_ALERT_3_G Average HKL Measurement Multiplicity ..........         4.4 Low
PLAT969_ALERT_5_G The 'Henn et al.' R-Factor-gap value ..........       3.079 Note
              Predicted wR2: Based on SigI**2  4.09 or SHELX Weight 12.24
PLAT978_ALERT_2_G Number C-C Bonds with Positive Residual Density.         10 Info
PLAT992_ALERT_5_G Repd & Actual _reflns_number_gt Values Differ by          2 Check
```

0 **ALERT level A** = Most likely a serious problem - resolve or explain
0 **ALERT level B** = A potentially serious problem, consider carefully
8 **ALERT level C** = Check. Ensure it is not caused by an omission or oversight
13 **ALERT level G** = General information/check it is not something unexpected

4 ALERT type 1 CIF construction/syntax error, inconsistent or missing data
5 ALERT type 2 Indicator that the structure model may be wrong or deficient
5 ALERT type 3 Indicator that the structure quality may be low
4 ALERT type 4 Improvement, methodology, query or suggestion
3 ALERT type 5 Informative message, check

It is advisable to attempt to resolve as many as possible of the alerts in all categories. Often the minor alerts point to easily fixed oversights, errors and omissions in your CIF or refinement strategy, so attention to these fine details can be worthwhile. In order to resolve some of the more serious problems it may be necessary to carry out additional measurements or structure refinements. However, the purpose of your study may justify the reported deviations and the more serious of these should normally be commented upon in the discussion or experimental section of a paper or in the "special_details" fields of the CIF. checkCIF was carefully designed to identify outliers and unusual parameters, but every test has its limitations and alerts that are not important in a particular case may appear. Conversely, the absence of alerts does not guarantee there are no aspects of the results needing attention. It is up to the individual to critically assess their own results and, if necessary, seek expert advice.

Publication of your CIF in IUCr journals

A basic structural check has been run on your CIF. These basic checks will be run on all CIFs submitted for publication in IUCr journals (*Acta Crystallographica*, *Journal of Applied Crystallography*, *Journal of Synchrotron Radiation*); however, if you intend to submit to *Acta Crystallographica Section C* or *E* or *IUCrData*, you should make sure that full publication checks are run on the final version of your CIF prior to submission.

Publication of your CIF in other journals

Please refer to the *Notes for Authors* of the relevant journal for any special instructions relating to CIF submission.

PLATON version of 13/05/2024; check.def file version of 04/05/2024

Datablock 1 - ellipsoid plot

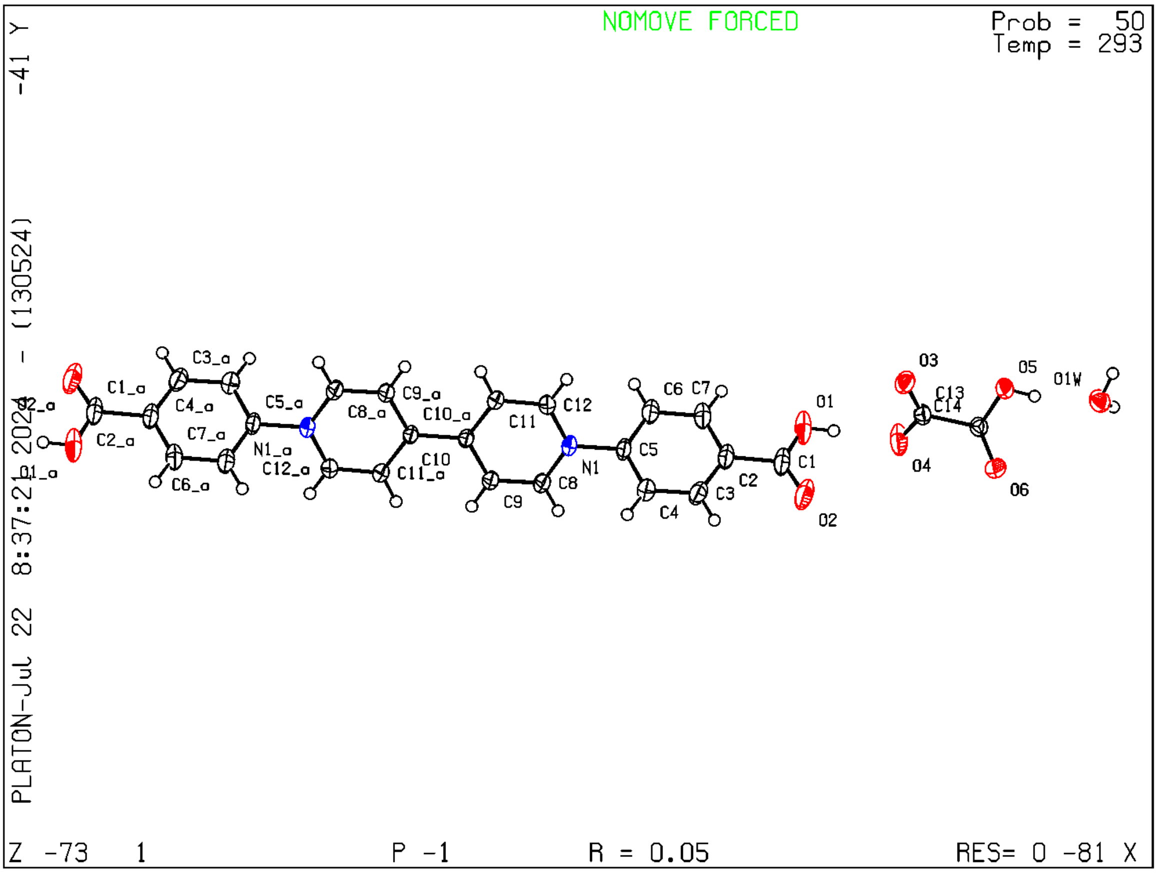

Supplement: Supplementary file 2 — Supporting Information [file ADVS-13-e18602-s001.zip › checkcif-ok.pdf]
